# Seasonal patterns of oxidative stress markers in captive Asian elephants in Thailand and relationships to elephant endotheliotropic herpesvirus shedding

**DOI:** 10.3389/fvets.2023.1263775

**Published:** 2023-09-19

**Authors:** Worapong Kosaruk, Janine L. Brown, Patcharapa Towiboon, Kidsadagon Pringproa, Veerasak Punyapornwithaya, Pallop Tankaew, Narueporn Kittisirikul, Wachiraporn Toonrongchang, Thittaya Janyamathakul, Panida Muanghong, Chatchote Thitaram

**Affiliations:** ^1^Doctoral Degree Program in Veterinary Science, Faculty of Veterinary Medicine, Chiang Mai University, Chiang Mai, Thailand; ^2^Center of Elephant and Wildlife Health, Chiang Mai University Animal Hospital, Chiang Mai, Thailand; ^3^Elephant, Wildlife, and Companion Animals Research Group, Chiang Mai University, Chiang Mai, Thailand; ^4^Center for Species Survival, Smithsonian Conservation Biology Institute, Front Royal, VA, United States; ^5^Department of Veterinary Bioscience and Veterinary Public Health, Faculty of Veterinary Medicine, Chiang Mai University, Chiang Mai, Thailand; ^6^Central Laboratory, Faculty of Veterinary Medicine, Chiang Mai University, Chiang Mai, Thailand; ^7^Elephant Hospital, National Elephant Institute, Forest Industry Organization, Lampang, Thailand; ^8^Baanchang Elephant Park, Mae Thang, Chiang Mai, Thailand; ^9^Pattara Elephant Farm, Chiang Mai, Thailand; ^10^Mae Taeng Elephant Park and Clinic, Chiang Mai, Thailand; ^11^Department of Companion Animal and Wildlife Clinic, Faculty of Veterinary Medicine, Chiang Mai University, Chiang Mai, Thailand

**Keywords:** Asian elephants, seasonal pattern, oxidative stress markers, stress markers, elephant endotheliotropic herpesvirus shedding

## Abstract

**Introduction:**

Oxidative stress refers to an imbalance between oxidant and antioxidant activity and accumulation of reactive oxygen species, which can have detrimental effects on animal health. Annual fluctuations in oxidative stress status can occur, increasing disease susceptibility during certain time periods. However, a full understanding of factors related to oxidative stress in Asian elephants and how to mitigate the negative consequences is lacking.

**Methods:**

This study measured six serum oxidative stress markers [reactive oxygen species (ROS), malondialdehyde (MDA), 8-hydroxydeoxyguanosine (8-OHdG), albumin, glutathione peroxidase (GPx), and catalase] and two stress markers [serum cortisol and fecal glucocorticoid metabolites (fGCM)] in 23 captive Asian elephants in Thailand over a 12 months period to examine relationships with age and season.

**Results:**

Seasonal variations were observed, with several markers exhibiting significantly higher concentrations in the summer (ROS, MDA, 8-OHdG, albumin) and lower values during the rainy/winter seasons (MDA, 8-OHdG, albumin, catalase). By contrast, GPx was the only marker to be highest during the rainy season. For the stress markers, higher fGCM concentrations were noted during the rainy season, which contrasts with earlier studies showing more activity in the winter (tourist season). Positive correlations were found between the temperature-humidity index and ROS, GPx, and fGCM, while a negative correlation was observed with serum albumin. Elephant endotheliotropic herpesvirus (EEHV) shedding events were associated with higher concentrations of ROS and MDA. A moderate negative correlation was observed between 8-OHdG and the PCR threshold cycle of EEHV shedding (Ct), indicating DNA damage may be involved in EEHV shedding in elephants.

**Discussion:**

Results revealed significant age and seasonal effects on several oxidative stress markers, indicating those factors should be considered in study design and data interpretation. There also may be physiological adaptations in oxidative stress conditions in relation to environmental changes that could impact health outcomes.

## 1. Introduction

The Asian elephant (*Elephas maximus*) is the national symbol of Thailand and plays an important role in animal-related ecotourism throughout the country (1, 2). Most captive elephants in Thailand are privately owned and housed in elephant facilities (or camps) that vary greatly in management due to lack of official husbandry standards or welfare guidelines (3). As such, elephant welfare varies among camps and tourist activities, leading to varied effects on health and disease status (1, 4, 5). According to records from the elephant hospital in Lampang (Thai Elephant Conservation Center, Forest Industry Organization of Thailand) between November 2019 and May 2022, the main health disorders of elephants were wounds (25.7%) and gastrointestinal distress (23.5%). Although infectious diseases accounted for only 5.4% of all cases, some had high fatality. For example, elephant endotheliotropic herpesvirus-hemorrhagic disease (EEHV-HD) was responsible for 3.2% of the infectious disease cases during that time period, with a mortality rate of 86%. The virus is endemic in elephants; adults shed it intermittently, with calves under 8 years of age being most susceptible to clinical disease (6–10), leading to difficulty in disease control (10–12). EEHV-HD may be associated with changes in oxidative stress markers (6, 7, 9) comparable to herpesvirus infections in mammals and birds (13). Recently, lower albumin and higher malondialdehyde (MDA), glutathione peroxidase (GPx) and catalase activities were found in EEHV-infected elephant calves (14).

Oxidative stress is a pathological condition defined as an imbalance between oxidative molecules and antioxidants, and whereby the body cannot cope with excess free radical production (i.e., reactive oxygen species; ROS) leading to oxidative damage and cell death (13). Reports in humans and animals have shown that oxidative stress is involved in disease susceptibility and progression (13, 15). For example, it increases the risk of myocardial injury in chronic renal failure (16) and mortality associated with infectious hemorrhagic-related diseases (e.g., leptospirosis, dengue, and malaria) (17–19), and has been linked to herpesvirus reactivation from latency (20). Oxidative stress is induced by physiological and pathological conditions such as trauma, aging, cancer, pregnancy, and infection (21). A meta-analysis conducted by Sebastiano et al. (13) provided evidence that oxidative stress may selectively enhance the susceptibility and progression of herpesvirus infection in vertebrates. In elephants, oxidative stress markers are influenced by age, season, and disease conditions, with acute EEHV-HD exhibiting the most significant changes (14). Physiological stress (i.e., increased glucocorticoid concentrations) also can contribute to oxidative stress in many species (22, 23). However, oxidative imbalances are not always obvious based on general appearances or standard hematology tests (15). Hence, measurement of specific markers to assess oxidative stress status could significantly advance our knowledge of disease states and development of effective treatment therapies.

Several biological markers have been used as indicators of oxidative stress, such reactive oxygen species (ROS), MDA, 8-hydroxydeoxyguanosine (8-OHdG), serum albumin, and enzymatic antioxidants (e.g., GPx and catalase) (14, 24, 25). ROS are endogenously produced free radicals that are associated with a wide variety of clinical disorders, including cardiovascular disease, diabetes, cancer, and infections (26). MDA is a byproduct of lipid peroxidation that is highly toxic to cell membranes (27). In humans, herpesvirus-infected patients were found to have significant changes in serum MDA concentrations during periods of disease activity and remission (28), as were EEHV-HD cases in Asian elephants (14). 8-OHdG is a marker for oxidative DNA damage and has been used to monitor and predict clinical outcomes in several conditions, such as renal failure (29), cancer (30), and herpesvirus infections (31). Serum albumin has antioxidant properties and serves as an important antioxidant in extravascular fluids (32). GPx and catalase are enzymatic antioxidants that play fundamental roles in breaking down hydrogen peroxide (the primary contributor of oxidative molecules) to water. Decreased activity of both enzymes has been associated with viral infections, metabolic disorders, and degenerative diseases (33, 34). Thus, assessing these oxidative markers could provide useful information in the health monitoring of elephants.

Previous studies have identified seasonal variation in oxidative stress in several species as part of normal physiological responses to cope with environmental change (35–37). Changes in oxidative stress markers associated with the temperature-humidity index (THI) have been demonstrated in several species; for example, higher oxidant (ROS and MDA) and antioxidant (GPx and catalase) markers were observed during hot summer months in cattle in south Asia as a response to heat stress (38–40). In Thailand, Yun et al. (10) reported an influence of season on the frequency of EEHV-HD in captive elephant calves, with the highest number of cases found during the rainy season; however, data on whether shedding also is related to oxidative stress status are lacking. Thus, this study aimed to investigate annual patterns of several serum oxidative stress biomarkers in captive Asian elephants in Thailand, and correlate those to levels of EEHV shedding. Understanding how oxidative stress and viral shedding are influenced by seasonal changes might help veterinarians understand more about the factors responsible for and pathogenesis of this deadly disease in elephants.

## 2. Materials and methods

### 2.1. Animal ethical consent

Animal ethics approval was obtained from the Animal Care and Use Committee, Faculty of Veterinary Medicine, Chiang Mai University (FVM, CMU), reference number S7/2564.

### 2.2. Elephants and sample collection

The study was conducted from June 2021 to September 2022. Twenty-three captive Asian elephants at seven tourist camps in Thailand (Chiang Mai and Lampang provinces) participated in the study (mean age, 14.2 ± 14.0 years; range, 1–50 years). Elephants were categorized into two groups according to EEHV susceptibility risk (12): calves (≤8 years, E1–E12, mean age, 5.0 ± 2.3 years, range 21 months–8 years; male = 5, female = 7) and adults (>8 years, E13–E23, mean age, 24.3 ± 14.5 years, range 10–50 years; male = 1, female = 10). Although housed at tourist camps, none of the elephants participated in tourist activities during this study because of an international travel ban due to the COVID-19 pandemic. Information on camps and previous EEHV-HD history is presented in Table 1. Elephants were fed mainly fresh roughage (e.g., Napier grass, corn stalks), with high energy supplements (e.g., bananas, sugar cane) provided occasionally. Four elephants had previously survived an episode of EEHV-HD Type 1 (E11, E12, and E13) and Type 3/4 (E9). Each had been transported to the elephant hospital at the Thai Elephant Conservation Center (Lampang, Thailand) and provided intensive care (fluid therapy, supportive treatment, and antiviral drugs), after which they returned to their respective elephant camps. General information on the EEHV-HD cases, including age, sex, camp, year of active EEHV-HD, and initial clinical signs is shown in Supplementary Table S1.

**Table 1:** General information about the number of elephants and previous EEHV-HD history in the seven camps of this study.

Camp	Location	Total number of elephants	Number of study elephants	Previous EEHV-HD cases	EEHV type detected	Comments
A	Chiang Mai	52	4	None	–	Located near Camp B
B	Chiang Mai	30	3	2013, 2017	1A and 1B	Died (2013); survived (2017)
C	Chiang Mai	35	7	2022	1A	Survived
D	Chiang Mai	15	1	2021	1A	Survived
E	Chiang Mai	27	2	2015, 2017	3/4 and 1A + 3/4	Died
F	Chiang Mai	6	1	2018	4	Survived
G	Lampang	31	5	2014, 2018, 2019	1A	Survived (2014, 2019); died (2018)

EEHV-HD, elephant endotheliotropic herpesvirus-hemorrhagic disease; EEHV, elephant endotheliotropic herpesvirus.

Blood, buccal and fecal samples were collected approximately monthly from each elephant in the morning (9.00 to 12.00 h) and transported in a cool box to the laboratory at FVM, CMU. Blood (~5 mL, *N* = 251) collected from an ear vein using a 21G scalp vein needle attached to a 5 mL syringe was transferred to a red-top serum tube (BD Vacutainer^®^ Serum, Franklin Lakes, NJ, United States) and allowed to clot at room temperature for 1 h for assessment of oxidative stress markers. Buccal swab samples (*N* = 238) were collected using a sterile nylon swab and placed into a sterile tube containing 1 mL of phosphate buffer solution to quantify EEHV shedding. Fresh fecal samples (20 g, *N* = 248) were collected into a zip-lock bag on the same day as the swab and blood samples for measuring fecal glucocorticoid metabolites (fGCM). Blood was centrifuged (Hettich, Westphalia, Germany) at 700 × g for 10 min and the serum was stored at −80°C until analysis. Buccal swab samples were kept in a 4°C refrigerator and processed within 24 h. Fecal samples were stored at −20°C until extraction and subsequent analysis.

### 2.3. Oxidant markers

ROS and MDA were measured in serum samples following validated protocols for Asian elephants (14). Concentrations of 8-OHdG were measured by an oxidative DNA damage enzyme immunoassay kit (Cat #K059-H5, Arbor Assays, Michigan, United States) validated for Asian elephant serum by demonstrating parallelism between serial dilutions of serum and the standard curve (*y* = −0.0093*x* + 82.74, *R*^2^ = 0.86) and a significant recovery of 8-OHdG added to a low concentration sample before analysis (*y* = 0.9902*x* + 122.47, *R*^2^ = 0.99). Serum was diluted 1:10 for analysis, absorbance was measured at 450 nm in a microplate reader (TECAN, Männedorf, Switzerland), and concentrations were expressed as ng/ml. Assay sensitivity was 0.072 ng/mL. Intra-and inter-assay coefficients of variation based on concentration were 6.3 and <10%, respectively.

### 2.4. Antioxidant markers

Serum GPx and catalase activities were quantified based on protocols validated for Asian elephant serum (14). Serum albumin was quantified by using an automated chemistry analyzer (BX-3010, Sysmex Corporation, Tokyo, Japan) according to the manufacturer’s protocol.

### 2.5. Stress markers

Serum was ether extracted under a fume hood for analysis of cortisol. Briefly, 150 μL of serum was added to diethyl ether (600 μL, RCI Labscan) and vortexed for 30 s. The solvent layer was allowed to separate for 5 min at room temperature and then placed in a dry ice ethanol bath until the serum fraction was completely frozen (10 s). Ether was decanted from the frozen serum into a new glass tube and evaporated off in a heat block (60°C, 5–10 min). Samples were resuspended in 150 μL of assay buffer and stored at −20°C until analysis. Cortisol concentrations were measured by a double-antibody enzyme immunoassay (EIA) using a secondary goat anti-rabbit IgG antibody and polyclonal rabbit anti-cortisol antibody (R4866, Coralie Munro, University of California Davis, CA, United States) validated for elephants (41). The assay was validated by demonstrating serial dilutions of ether-extracted serum pools were parallel to the standard curve (*y* = −39.54*x* ± 70.011, *R*^2^ = 0.89). Recovery of serum cortisol concentrations added to a low-concentration sample before analysis was significant (*y* = 0.9604*x* + 0.0523, *R*^2^ = 0.99). Extracted serum was analyzed in duplicate (neat to 1:4) and absorbance measured at 450 nm. Assay sensitivity was 0.11 ng/mL. The intra-and inter-assay coefficients of variation were <10 and 9.3%, respectively.

Fecal sample extraction and analysis followed Kosaruk et al. (2). Briefly, frozen samples were thawed at room temperature before drying in a conventional oven at 60°C for 24–48 h. Dried fecal powder (0.1 g ± 0.01 g) was extracted by adding 5 mL of 90% EtOH, vortexing briefly (10 s), and boiling in a water bath (90°C) for 20 min. Additional 95% EtOH was added to maintain the volume at 5 mL. After boiling, the tubes were centrifuged at 960 × g for 20 min and the supernatants poured into new tubes. Fecal pellets were extracted again and the supernatants combined, dried in a 90°C water bath, resuspended in 3 mL of 95% EtOH, and dried again. Final extracts were resuspended in 1 mL of 50% methanol and stored at −20°C until analysis. Fecal extracts were diluted 1:3 in assay buffer (0.0137 M Trizma base, 0.2 M Triz-HCl, 0.2 M NaCl, 0.2 M EDTA, 0.001% BSA, and 0.001% Tween 20; pH 7.5) and fGCM concentrations measured by double-antibody EIA with a polyclonal rabbit anti-corticosterone antibody (CJM006, Coralie Munro) validated for Asian elephants in Thailand (42). Samples and corticosterone standards (50 μL) were added to wells in duplicate followed by corticosterone-HRP (25 μL; 1:30,000) and anti-corticosterone antibody (25 μL; 1:100,000). Plates were incubated in the dark at room temperature for 2 h before adding 100 μL of TMB solution, followed by incubation for 20–35 min, and then addition of stop solution (50 μL). Absorbance was measured at 450 nm by a microplate reader (TECAN). Assay sensitivity was 0.192 ng/g, and intra-and inter-assay coefficients of variation based on concentration were <10 and 11.33%, respectively.

### 2.6. EEHV shedding analysis

Buccal samples were vortexed for 30 s and centrifuged at 700 × g for 10 min. An 200 μL aliquot of the supernatant was gently removed for DNA extraction using a commercial kit (NucleoSpin^®^ Blood, MACHEREY-NAGEL Inc., Allentown PA, United States). Extracted DNA samples were stored at −20°C until analysis. The real-time polymerase chain reaction (PCR) was performed following the protocol of Stanton et al. (43) for assessing EEHV 1 and EEHV 3/4 (Pacific Science CO., LTD., Bangkok, Thailand) (44–46). The Asian elephant tumor necrotic factor gene was used as an internal control (TNF, Pacific Science CO., LTD., Bangkok, Thailand). Samples were considered positive when the threshold cycle (EEHV Ct) was between 20 and 40, and the negative control (sterile water) Ct was 0 (46). Standard curves for EEHV1 (*R*^2^ = 0.99) and EEHV3/4 (*R*^2^ = 0.99) were constructed followed Stanton et al. (47) and used to quantify EEHV viral load (viral genome copies/mL or vgc/mL).

### 2.7. Seasonal and environmental determinations

The three major seasons in Thailand are winter (16 October–15 February), summer (16 February–15 May), and rainy (16 May–15 October) (Thai Meteorological Department, www.tmd.go.th (accessed on 16 May 2023)). THI was calculated following Yeotikar et al. (48) and presented in Supplementary Figure S1.

### 2.8. Statistical analysis

All data were analyzed by using R statistical software (RStudio, version 4.1.0). Descriptive data are presented as the mean ± standard deviation (SD) for each biomarker. A generalized least square model (GLS function; R package: non-linear mixed effect model (nlme) 3.1–148 (49)) was used to determine differences in means of biomarkers among age groups and months, followed by Tukey *Post Hoc* tests. Assumptions of GLS including normality and homogeneity of variance of the residuals were assessed by examining the normal Q–Q plot and residuals vs. fitted values plots, respectively. If the assumptions were met, the biomarker concentrations were analyzed without any additional transformations. Repeated measures correlations were then used to determine relationships between each biomarker, THI, and EEHV shedding (Ct and viral load) data.

## 3. Results

Summaries of monthly means (±SD) for oxidant (serum ROS, MDA, 8-OHdG), antioxidant (serum albumin, GPx, catalase) and stress (serum cortisol, fGCM) markers are presented in Table 2 with data according to the three major seasons in Thailand shown in Table 3.

**Table 2:** Biomarker concentrations (means ± SD) and ranges according to three seasons in Thailand.

Biomarkers	Winter (16 October–15 February) *N* = 79	Summer (16 February–15 May) *N* = 63	Rainy (16 May–15 October) *N* = 110
ROS (mg/L)	2.39 ± 0.08 (2.14–2.57)	2.46 ± 0.13 (2.22–2.77)	2.42 ± 0.09 (2.18–2.62)
MDA (nmol/mL)	1.79 ± 0.27 (1.04–2.99)	1.86 ± 0.27 (1.34–2.99)	1.66 ± 0.34 (1.04–2.69)
8-OHdG (ng/mL)	7.31 ± 1.86 (3.79–13.53)	8.63 ± 2.15 (5.25–14.33)	7.31 ± 1.86 (3.73–15.24)
Albumin (g/dL)	3.40 ± 0.27 (2.80–4.00)	3.46 ± 0.22 (2.90–3.90)	3.25 ± 0.24 (2.80–3.80)
GPx (U/L)	1.34 ± 0.45 (0.28–2.51)	1.26 ± 0.58 (0.24–2.90)	1.77 ± 0.79 (0.23–3.99)
Catalase (U/mL)	11.91 ± 5.47 (3.29–24.61)	16.51 ± 5.53 (7.55–38.76)	15.10 ± 6.93 (4.72–32.10)
Serum cortisol (ng/mL)	2.79 ± 1.81 (0.29–9.45)	2.80 ± 1.51 (0.34–7.96)	2.86 ± 1.57 (0.44–9.14)
fGCM (ng/g)	50.18 ± 15.19 (22.15–104.36)	55.85 ± 14.60 (23.91–86.11)	63.21 ± 15.46 (23.80–106.08)

ROS, reactive oxygen species; MDA, malondialdehyde; 8-OHdG, 8-hydroxydeoxyguanosine; GPx, glutathione peroxidase; fGCM, fecal glucocorticoid metabolites.

**Table 3:** Overall means (±SD) and range of monthly oxidative, antioxidative, and stress biomarker concentrations for all elephants combined.

Biomarkers	Jan	Feb	Mar	Apr	May	Jun	Jul	Aug	Sep	Oct	Nov	Dec
ROS (mg/L)	2.36 ± 0.10 (2.14–2.51)	2.41 ± 0.15 (2.22–2.77)	2.31 ± 0.09 (2.26–2.62)	2.24 ± 0.10 (2.23–2.69)	2.28 ± 0.10 (2.18–2.61)	2.34 ± 0.11 (2.20–2.56)	2.44 ± 0.12 (2.34–2.56)	2.42 ± 0.06 (2.25–2.52)	2.40 ± 0.07 (2.30–2.55)	2.40 ± 0.06 (2.26–2.51)	2.36 ± 0.06 (2.23–2.45)	2.45 ± 0.09 (2.27–2.57)
MDA (nmol/mL)	1.75 ± 0.27 (1.04–2.09)	1.76 ± 0.15 (1.64–2.24)	1.90 ± 0.37 (1.34–2.99)	1.92 ± 0.23 (1.49–2.54)	1.85 ± 0.25 (1.49–2.54)	1.52 ± 0.47 (1.04–2.69)	1.61 ± 0.30 (1.04–2.24)	1.51 ± 0.28 (1.04–2.39)	1.82 ± 0.18 (1.49–2.24)	1.72 ± 0.19 (1.49–2.24)	1.89 ± 0.37 (1.34–2.99)	1.81 ± 0.24 (1.34–2.24)
8-OHdG (ng/mL)	7.60 ± 2.11 (4.95–13.16)	8.70 ± 2.30 (5.55–14.33)	8.78 ± 2.17 (5.27–13.77)	8.40 ± 2.06 (5.25–13.34)	9.39 ± 1.91 (6.57–14.66)	8.61 ± 2.14 (6.12–15.23)	8.01 ± 2.02 (5.15–13.95)	7.11 ± 1.91 (3.73–11.62)	6.79 ± 1.78 (4.60–11.52)	7.00 ± 2.00 (4.38–11.98)	6.96 ± 0.88 (5.86–8.60)	7.64 ± 2.10 (3.79–13.52)
Albumin (g/dL)	3.37 ± 0.28 (2.80–3.90)	3.35 ± 0.26 (2.90–3.70)	3.51 ± 0.19 (3.20–3.90)	3.51 ± 0.18 (3.30–3.80)	3.49 ± 0.22 (3.00–3.80)	3.26 ± 0.15 (2.80–3.50)	3.26 ± 0.25 (2.80–3.80)	3.08 ± 0.18 (2.80–3.40)	3.13 ± 0.17 (2.80–3.40)	3.30 ± 0.31 (2.90–4.00)	3.47 ± 0.27 (3.00–4.00)	3.45 ± 0.23 (2.90–3.80)
GPx (U/l)	1.32 ± 0.27 (0.87–2.06)	1.28 ± 0.52 (0.24–2.11)	1.40 ± 0.75 (0.25–2.90)	1.11 ± 0.41 (0.48–2.22)	1.78 ± 0.47 (0.35–2.42)	2.11 ± 0.84 (0.48–3.99)	2.09 ± 0.88 (0.50–3.91)	1.79 ± 0.82 (0.59–3.10)	1.08 ± 0.39 (0.23–1.68)	1.13 ± 0.38 (0.28–1.95)	1.66 ± 0.46 (0.95–2.51)	1.26 ± 0.53 (0.51–2.22)
Catalase (U/mL)	16.68 ± 4.63 (7.18–24.61)	16.45 ± 4.87 (8.80–25.15)	18.36 ± 6.53 (8.73–38.76)	14.71 ± 4.62 (7.55–26.45)	13.93 ± 5.34 (6.52–25.50)	18.05 ± 7.01 (7.95–28.09)	19.81 ± 7.02 (8.11–31.55)	13.09 ± 6.15 (4.72–32.10)	10.22 ± 4.57 (5.44–21.43)	7.16 ± 1.62 (3.29–9.46)	8.81 ± 4.08 (4.15–20.19)	14.40 ± 4.33 (8.03–21.29)
Serum cortisol (ng/mL)	3.15 ± 1.82 (0.45–9.45)	2.32 ± 1.06 (0.92–5.49)	3.10 ± 1.81 (0.34–7.57)	2.99 ± 1.50 (0.98–7.96)	3.05 ± 1.50 (0.45–5.82)	2.80 ± 1.51 (0.83–6.11)	2.91 ± 1.41 (0.85–6.21)	2.96 ± 2.02 (0.99–9.14)	2.56 ± 1.39 (0.44–5.07)	2.19 ± 1.29 (0.64–5.06)	2.61 ± 1.67 (0.29–6.51)	3.20 ± 2.26 (0.74–8.50)
fGCM (ng/g)	51.76 ± 12.41 (34.85–81.16)	50.14 ± 13.20 (23.91–71.81)	49.18 ± 10.48 (30.65–63.06)	67.94 ± 11.92 (46.09–86.11)	60.19 ± 16.87 (37.30–94.68)	68.63 ± 15.65 (45.93–106.08)	62.39 ± 16.04 (44.10–97.87)	56.08 ± 13.06 (23.80–83.14)	68.46 ± 12.48 (49.99–88.79)	51.87 ± 17.31 (30.07–104.36)	43.68 ± 13.83 (22.15–74.11)	53.16 ± 16.18 (33.76–91.07)

ROS, reactive oxygen species; MDA, malondialdehyde; 8-OHdG, 8-hydroxydeoxyguanosine; GPx, glutathione peroxidase; fGCM, fecal glucocorticoid metabolites.

### 3.1. Oxidant markers

**Figure 1:**
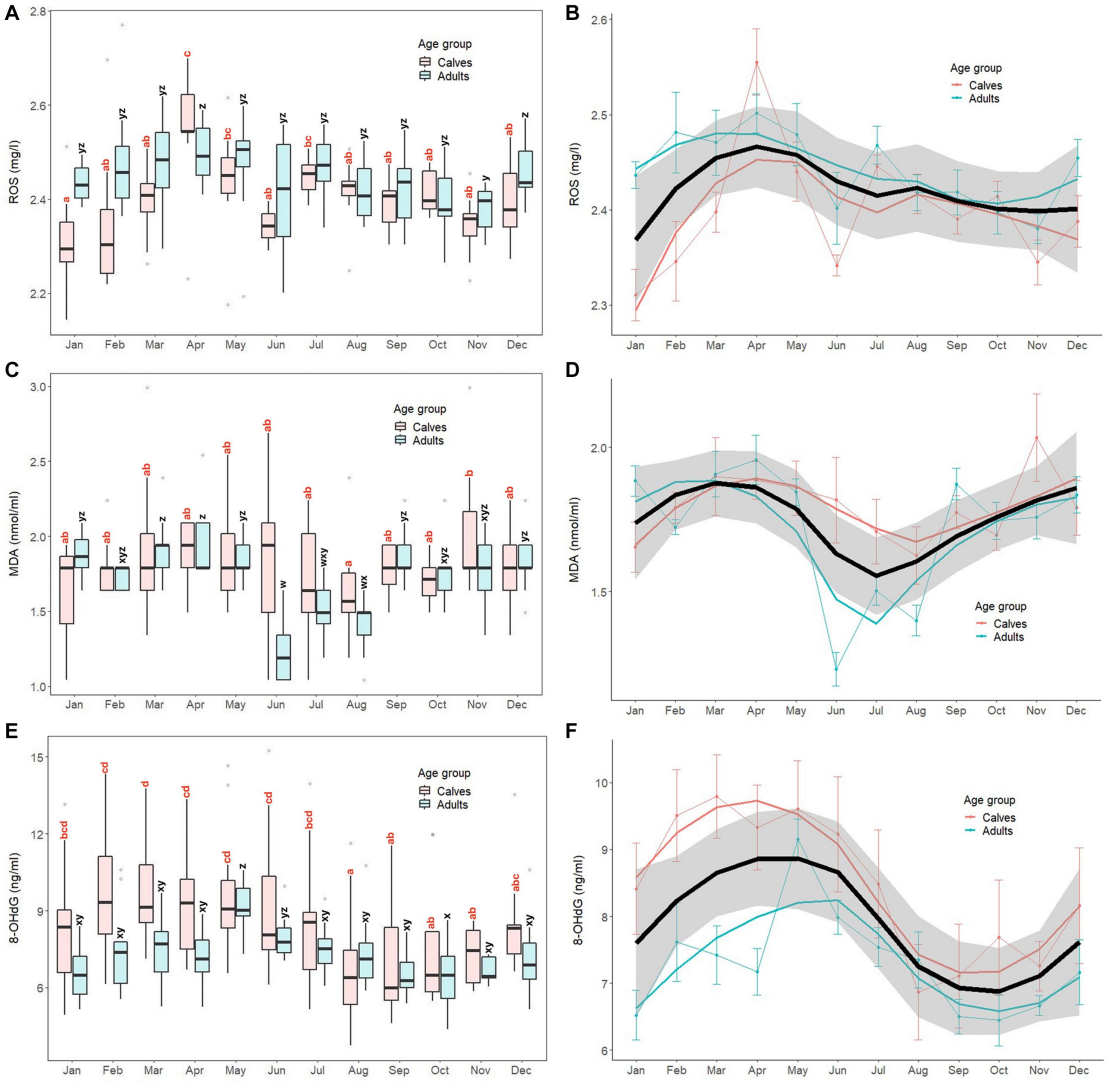
Box and line plots display monthly patterns of serum oxidant marker concentrations in calves (≤8 years old, *N* = 12) and adult elephants (>8 years, *N* = 11): reactive oxygen species, ROS (**A, B**); malondialdehyde; MDA (**C, D**); 8-hydroxydeoxyguanosine; 8-OHdG (**E, F**). Boxplots represent median, quartiles, and the 25th/75th percentiles, error bars represent the 10th/90th percentiles, and open circles indicate outliers. Different superscripts show a significant month effect (*p* < 0.05) for calves (*a*,*b*,*c*,*d*) and adults (*w*,*x*,*y*,*z*). Bold black lines represent the overall mean trend line, and the shaded area is the 95% confidence interval. Pink solid lines represent the trendline for calves (*N* = 12), solid blue lines represent the trendline for adults (*N* = 11), and thin lines with dots represent monthly means (±SD) in each age group (pink: calves, blue: adults).

Monthly patterns of oxidant marker (serum ROS, MDA, 8-OHdG) concentrations in calves and adult elephants are shown in Figure 1, with results of GLS analyses for all elephants combined presented in Supplementary Table S2. For ROS, concentrations fluctuated throughout the year, with generally higher concentrations in the summer and lower concentrations in the winter (Figures 1A,B). For calves, the highest concentration was observed in April, with the lowest in January. In adults, ROS concentrations were more stable compared to calves, but followed a similar pattern, with a peak in April similar to calves, and again in December, with the lowest concentrations exhibited in November. The GLS analysis (Supplementary Table S3) revealed ROS concentrations were higher in adults (2.44 ± 0.09 mg/L) than calves (2.40 ± 0.10 mg/L, *p* < 0.01). Compared to the reference value in January, ROS was higher in summer (March to May), rainy (July to October), and winter (December) months for all animals combined. For interactions, there were differences in adults during April (2.19 ± 0.05 mg/L), July (2.47 ± 0.06 mg/L), August (2.42 ± 0.06 mg/L), September (2.42 ± 0.08 mg/L), and October (2.40 ± 0.07 mg/L). Compared to the reference value (calves × January; 2.31 ± 0.09 mg/L, *p* < 0.01).

For MDA (Figures 1C,D), overall concentrations trended higher in late winter and summer and lower in the mid rainy season, more so for adults than calves, the latter of which were more stable across the year. In calves, the lowest concentrations were observed in August, while the highest were in November, with both being significantly different (*p* < 0.05). By contrast, in the adult group, there was a notable decrease in MDA concentrations during rainy season months (June, July, August), with the lowest in June. Based on the GLS analysis (Supplementary Table S2), no differences were found between calves (1.79 ± 0.34 nmoL/mL) and adults (1.71 ± 0.29 nmoL/mL, *p* > 0.05). Overall MDA concentrations were higher in March, April, May, and November compared to the reference value (January, *p* < 0.05). Interaction effects were observed, with adults during June (1.23 ± 0.19 nmoL/mL), July (1.50 ± 0.17 nmoL/mL), August (1.40 ± 0.18 nmoL/mL), and November (1.76 ± 0.23 nmoL/mL) being different compared to the reference value (calves × January; 1.65 ± 0.30 nmoL/mL, *p* < 0.05).

For 8-OHdG (Figures 1E,F), the overall trend was for higher concentrations in the summer and lower concentrations in late rainy/early winter months, especially for calves. In that group, the lowest concentrations were found in August, which then remained relatively stable from September to November. Subsequently, concentrations increased, reaching a peak in March that was sustained for the following 4 months (April to July). In the adult group, concentrations were low from January to April, then increased sharply to a peak in May, gradually declining to low levels until the end of the year. GLS analysis (Supplementary Table S2) found 8-OHdG concentrations in calves (8.53 ± 2.50 ng/mL) were higher than those in adults (7.31 ± 1.35 ng/mL, *p* = 0.014). Overall, concentrations were higher in February, March, April, and May, and lower in August, September, and November compared to the reference value (January, *p* < 0.05). Few interactions were found, only adults in May (9.15 ± 1.00 ng/mL) and August (7.35 ± 1.40 ng/mL) compared to the reference value (calves × January; 8.41 ± 2.35 ng/mL).

### 3.2. Antioxidant markers

**Figure 2:**
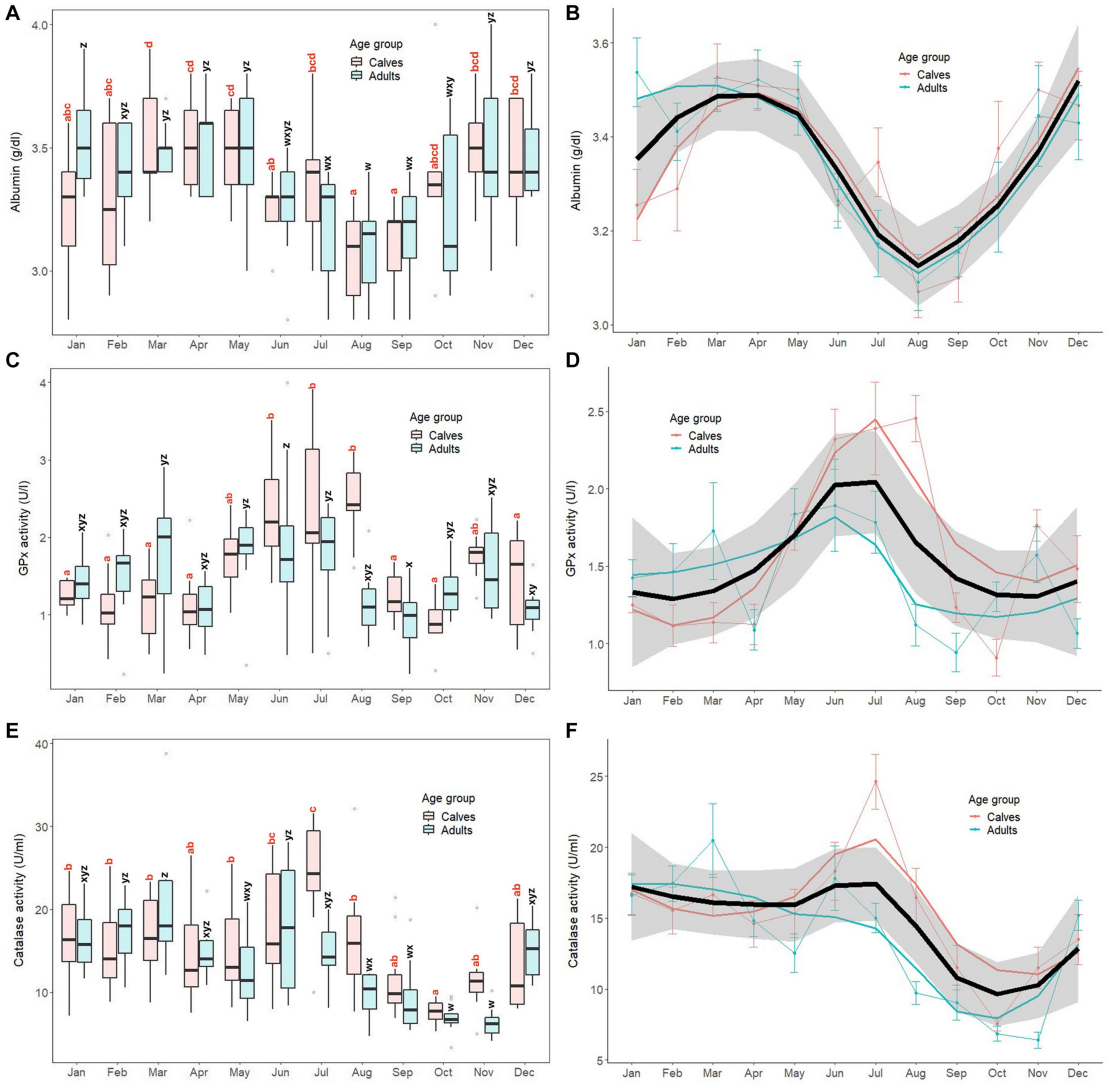
Box and line plots display monthly patterns of serum antioxidant marker concentrations in calves (≤8 years old, *N* = 12) and adult elephants (>8 years, *N* = 11): albumin (**A, B**); glutathione peroxidase; GPx (**C, D**); and catalase (**E, F**). Boxplots represent median, quartiles, and the 25th/75th percentiles, error bars represent the 10th/90th percentiles, and open circles indicate outliers. Different superscripts show a significant month variation at *p* < 0.05 within group; calves (*a*,*b*,*c*,*d*) and adults (*w*,*x*,*y*,*z*). Bold black lines represent the overall mean trend line, and the shaded area is the 95% confidence interval. Pink solid lines represent the trendline for calves (*N* = 12), solid blue lines represent the trendline for adults (*N* = 11), and thin lines with dots represent monthly means (±SD) in each age group (pink: calves, blue: adults).

Monthly patterns of antioxidant markers (serum albumin, GPx, catalase) in calves and adult elephants are shown in Figure 2, with results of GLS analyses for all elephants combined presented in Supplementary Table S3. For serum albumin, clear seasonal patterns were similar in both age groups, with higher concentrations observed during summer and winter, and lower concentrations during the rainy season (Figures 2A,B). In calves, albumin concentrations were higher during the three summer months (March, April, May), and again in November and December. Conversely, concentrations were lowest during the rainy season in June and August. Similarly, in adults, serum albumin concentrations were high during summer (March to May) and winter (November to February) months, and low during the rainy season (June to October), with the lowest concentrations observed in August similar to calves. In the GLS analysis (Supplementary Table S3), adults (3.35 ± 0.27 g/dL) had higher albumin concentrations than calves (3.34 ± 0.26 g/dL, *p* < 0.01). Overall, concentrations were higher in March, April, May, November, and December, and lower in August and September compared to the reference value (January, *p* < 0.05). Several interaction effects were found, with differences in adults during March (3.49 ± 0.10 g/dL), April (3.52 ± 0.18 g/dL), May (3.48 ± 0.26 g/dL), June (3.26 ± 0.19 g/dL), July (3.17 ± 0.23 g/dL), November (3.44 ± 0.34 g/dL), and December (3.43 ± 0.25 g/dL) compared to the reference value (calves × January; 3.25 ± 0.26 g/dL, *p* < 0.05).

For GPx, activity was notably higher in the rainy season (Figures 2C,D). In calves, concentrations were fairly stable for most of the year, except 3 months during the rainy season (June, July, August) where higher activity was displayed. The GPx pattern in adults displayed more fluctuations throughout the year; concentrations peaked in June (mid rainy season), while the lowest activity was in September (late rainy season). GLS analysis (Supplementary Table S3) found no differences between calves (1.59 ± 0.73 U/L) and adults (1.44 ± 0.65 U/L, *p* > 0.05). GPx activity was higher in June, July, August, and November when compared to the reference value (January, *p* < 0.01). Few interactions were observed, with differences only in adults during July (1.78 ± 0.66 U/L) and August (1.12 ± 0.44 U/L) when compared to the reference value (calves × January; 1.25 ± 0.17 U/L, *p* < 0.05).

For catalase, the trend was lower concentrations in the late rainy and early winter months (Figures 2E,F). In calves, concentrations were highest in July (24.62 ± 6.39 U/mL) and lowest in October (7.56 ± 1.45 U/mL), otherwise remaining relatively stable. In adults, catalase activity also was lowest in October, plus November, with a higher concentration in March (20.45 ± 7.76 U/mL), although there was considerable fluctuation throughout the year. The GLS analysis (Supplementary Table S3) showed no differences between catalase concentrations in calves (15.52 ± 6.61 U/mL) and adults (13.40 ± 6.05 U/mL, *p* > 0.05). Higher catalase activities were found in July, while concentrations were lower in September, October, and November compared to the reference value (January, *p* < 0.01). Interactions were observed in adults in July (15.00 ± 3.42 U/mL) and August (9.74 ± 2.66 U/mL), compared to the reference value (calves × January; 16.73 ± 5.07 U/mL, *p* < 0.05).

### 3.3. Stress markers

**Figure 3:**
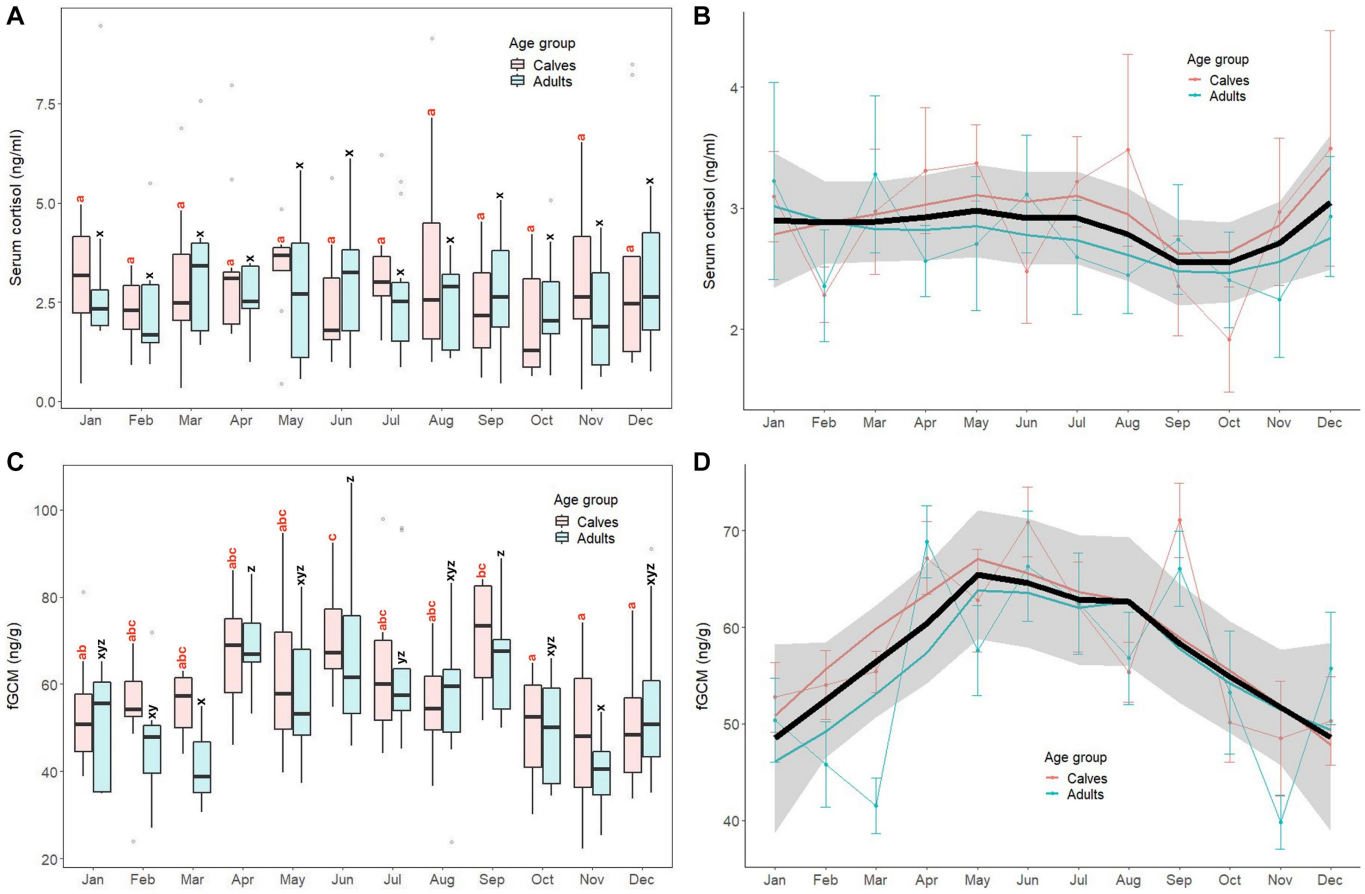
Box and line plots display monthly patterns of stress biomarkers in calves (≤8 years old, *N* = 12) and adult elephants (>8 years, *N* = 11): serum cortisol (**A, B**) and fecal glucocorticoid metabolites (**C, D**). Boxplots represent median, quartiles, and the 25th/75th percentiles, error bars represent the 10th/90th percentiles, and open circles indicate outliers. Different superscripts show a significant month variation at *p* < 0.05 within group; calves (*a*,*b*,*c*,*d*) and adults (*w*,*x*,*y*,*z*). Bold black lines represent the overall mean trend line, and the shaded area is the 95% confidence interval. Pink solid lines represent the trendline for calves (*N* = 12), solid blue lines represent the trendline for adults (*N* = 11), and thin lines with dots represent monthly means (±SD) in each age group (pink: calves, blue: adults).

Monthly patterns of stress markers (serum cortisol, fGCM) in calves and adult elephants are shown in Figure 3, with results of GLS analyses for all elephants combined presented in Supplementary Table S4. For serum cortisol (Figures 3A,B), concentrations were relatively stable throughout the year in both age groups. The GLS analysis (Supplementary Table S4) showed no differences in serum cortisol concentrations between calves (2.93 ± 1.69 ng/mL) and adults (2.72 ± 1.55 ng/mL). No month effect or interactions were found for this biomarker.

For fGCM, unlike serum cortisol, the overall trend was higher concentrations in summer and rainy seasons and lower concentrations in the winter (Figures 3C,D). In calves, the highest concentration was in June, with the lowest in October–December. In adults, concentrations were variable, with the highest concentrations in April, June and September, and lowest noted in both March and November. For the GLS analyses (Supplementary Table S4), there were no differences between calves (58.92 ± 14.92 ng/g) and adults (55.82 ± 17.19 ng/g, *p* > 0.05). Higher fGCM concentrations were found in April, May, June, and September when compared to the reference value (January, *p* < 0.01). No interactions were found (*p* > 0.05).

### 3.4. EEHV shedding

Data on EEHV shedding, including EEHV Ct and EEHV load are presented in Table 4, with annual patterns of biomarkers and EEHV shedding events for each individual elephant shown in Supplementary Figures S2–S9.

**Table 4:** Monthly shedding data of elephant endotheliotropic herpesvirus (EEHV) types EEHV1 or EEHV3/4 in participating elephant calves.

ID	Age	Sex	Camp	Month											
				Jan	Feb	Mar	Apr	May	Jun	Jul	Aug	Sep	Oct	Nov	Dec
E1	1y9m	M	B	iv	v/EEHV3/4 (23.25, 1,109,459)	v/neg	v/EEHV3/4 (29.69, 18,345)	v/neg	v/neg	v/neg	v/neg	v/neg	iv	v/EEHV3/4 (36.72, 208)	v/neg
E2	4y6m	F	C	iv	v/neg	v/EEHV1 (38.22, 9)	v/neg	v/neg	v/neg	v/neg	iv	NA	NA	NA	NA
E3	3y7m	M	C	iv	v/neg	iv	v/neg	v/neg	iv	v/neg	v/neg	v/neg	iv	iv	v/neg
E4	3y4m	F	C	iv	v/neg	v/neg	v/neg	NA	v/neg	v/neg	v/neg	iv	NA	NA	NA
E5	7y5m	M	A	v/neg	v/neg	v/neg	v/neg	v/neg	v/neg	v/neg	v/neg	iv	iv	iv	iv
E6	7y2m	F	A	iv	v/neg	NA	v/EEHV1 (29.11, 6,211)	v/neg	v/neg	NA	v/neg	iv	iv	iv	v/neg
E7	7y11m	F	A	iv	v/neg	v/neg	v/EEHV1 (28.04, 13,364)	v/neg	v/EEHV1 (31.31, 1,286)	v/neg	v/neg	iv	v/neg	v/neg	v/EEHV1 (35.65, 17,004)
E8	7y10m	F	E	iv	v/neg	NA	v/neg	v/EEHV1 (27.35, 21,902)	v/neg	v/neg	v/neg	v/neg	NA	v/EEHV1 (32.74, 39,156)	NA
**E9**	**5y4m**	**F**	**F**	**v/neg**	**v/EEHV3/4 (36.03, 323)**	**iv**	**v/EEHV3/4 (35.84, 365)**	**v/neg**	**NA**	**NA**	**NA**	**NA**	**NA**	**NA**	**NA**
E10	7y8m	F	E	iv	v/neg	NA	v/neg	v/neg	v/neg	v/neg	iv	v/EEHV3/4 (37.68, 113)	v/neg	v/neg	v/neg
**E11**	**7y10m**	**M**	**G**	**v/neg**	**v/EEHV1 (35.98, 45)**	**NA**	**v/neg**	**v/neg**	**v/neg**	**v/neg**	**v/neg**	**v/neg**	**iv**	**iv**	**v/neg**
**E12**	**2y10m**	**M**	**D**	**v/neg**	**v/neg**	**v/EEHV1 (34.15, 168)**	**v/EEHV1 (28.74, 8,096)**	**v/EEHV1 (35.59, 60)**	**v/neg**	**v/neg**	**v/neg**	**iv**	**v/neg**	**v/EEHV1 (37.84, 334)**	**iv**
**E13**	**9y11m**	**M**	**G**	**v/neg**	**v/neg**	**NA**	**v/neg**	**v/neg**	**v/EEHV1 (31.43, 1,180)**	**v/neg**	**v/EEHV1 (33.98, 190)**	**v/neg**	**iv**	**v/neg**	**v/neg**
E14	14y6m	F	G	v/neg	v/neg	v/neg	iv	v/neg	v/neg	v/neg	iv	v/neg	v/EEHV3/4 (38.09, 87)	v/EEHV3/4 (33.95, 1,216)	v/neg
E15	28y8m	F	G	v/neg	v/neg	v/neg	v/neg	v/neg	v/neg	iv	v/EEHV1 (39.22, 4)	v/neg	iv	v/neg	v/neg
E16	41y10m	F	G	iv	iv	v/neg	v/neg	v/neg	v/neg	v/neg	v/neg	v/neg	iv	NA	iv
E17	11y4m	F	C	v/neg	v/neg	v/neg	v/neg	NA	v/neg	iv	NA	v/neg	iv	v/neg	v/neg
E18	14y11m	F	C	iv	iv	NA	iv	v/neg	v/neg	v/neg	v/neg	v/neg	iv	v/neg	v/neg
E19	18y1m	F	C	NA	NA	NA	NA	v/neg	v/neg	v/neg	v/neg	v/neg	iv	NA	iv
E20	19y7m	F	C	iv	iv	v/neg	v/neg	v/neg	v/neg	v/neg	iv	iv	iv	v/neg	v/neg
E21	50y2m	F	B	iv	iv	v/neg	iv	v/neg	iv	v/neg	v/neg	v/neg	v/neg	iv	v/neg
E22	45y5m	F	B	NA	NA	NA	NA	v/neg	v/neg	v/neg	v/neg	v/neg	iv	NA	NA
E23	28y3m	F	A	v/neg	v/neg	v/neg	v/neg	v/neg	v/neg	v/EEHV3/4 (37.01, 173)	v/neg	iv	v/neg	v/neg	v/neg

Numbers in parentheses represent threshold cycles (Ct) and EEHV viral load as viral genome copies/mL. Data in bold represent shedding associated with EEHV-HD. Table modified from Ackerman et al. (45). iv, invalid sample, TNF not amplified; v, valid sample, TNF amplified; NA, no sample available; neg, EEHV not detected.

Eight elephants exhibited EEHV Type 1 shedding (34.8%), while five shed EEHV Type 3/4 (21.7%). No elephants shed both (i.e., no co-infection). EEHV shedding was observed in each month except January and generally not in consecutive months, with the exception of E12 that had 3 consecutive months of shedding (April–June) and E14 that shed Oct–Nov. Ten elephants never showed signs of EEHV shedding, although some samples were invalid due to lack of amplification, while five exhibited more than one episode during the study year. Shedders were generally younger than adults. Four male elephants (out of 6; 66.7%) and nine females (out of 17; 52.9%) were observed shedding. Shedders were observed at all seven camps, including Camp A that had no prior cases of EEHV-HD.

### 3.5. Correlation matrix

A correlation matrix showing relationships among oxidative and antioxidant stress makers, glucocorticoid stress markers, EEHV shedding, and THI data is shown in Table 5. For oxidative stress markers, weak positive correlations were found between albumin and ROS, MDA, and 8-OHdG concentrations. Moderate positive correlations were found between catalase and GPx, while ROS and 8-OHdG were weakly positively correlated to catalase. No other significant associations among oxidative stress markers were noted. For stress markers, only fGCM showed a weak positive correlation to 8-OHdG. Interestingly, serum cortisol and fGCM concentrations were not correlated. EEHV Ct was negatively correlated to 8-OHdG concentrations. A number of biomarkers were correlated to THI (ROS, GPx), including fGCM that was moderately positively correlated.

**Table 5:** Repeated measures correlations among oxidative stress status biomarkers, elephant endotheliotropic herpesvirus (EEHV) shedding, and the temperature-humidity index (THI).

Biomarkers		ROS	MDA	8-OHdG	Albumin	GPx	Catalase	Serum cortisol	fGCM	EEHV shedding		THI
										Ct	vgc/ml	
ROS												
MDA		0.099										
8-OHdG		0.066	0.055									
Albumin		0.144*	0.350**	0.215**								
GPx		−0.029	−0.016	0.051	−0.009							
Catalase		0.136*	0.067	0.198**	0.123	0.416**						
Serum cortisol		−0.027	0.049	−0.052	0.099	0.062	0.080					
fGCM		0.085	0.005	0.134*	−0.095	0.050	0.103	0.076				
EEHV Ct	−0.177	0.492	−0.608*	−0.140	0.362	−0.016	0.099	−0.441			
EEHV vgc/ml	−0.239	0.002	0.569	0.005	0.087	0.040	−0.104	0.192	−0.591*		
THI		0.195**	−0.073	0.117	−0.162*	0.229**	0.028	−0.004	0.334**	−0.157	−0.130	

ROS, reactive oxygen species; MDA, malondialdehyde; 8-OHdG, 8-hydroxydeoxyguanosine; GPx, glutathione peroxidase; fGCM, fecal glucocorticoid metabolites; EEHV Ct, threshold cycles; EEHV vgc, viral genome copies. Asterisks indicate significance differences at *p* < 0.05 (*) and *p* < 0.01 (**).

## 4. Discussion

This is the first study to investigate seasonal fluctuations of multiple oxidative stress biomarkers in captive Asian elephants in Thailand. Significant age-related effects were observed for ROS and albumin, which agrees with a previous study on oxidative stress markers in this population (14). Different annual patterns of each biomarker were found, demonstrating varied dependence on month or season. Based on overall trend lines, many of the oxidative stress markers had higher values in the summer months (ROS, MDA, 8-OHdG, albumin), with lower values in the rainy/winter seasons (MDA, 8-OHdG, albumin, catalase). GPx differed by having markedly higher activity in the rainy season. These variations imply physiological adaptations associated with environmental changes among these biomarkers that might have implications to disease susceptibility and recovery throughout the year. In the context of EEHV viral loads, we consider results preliminary owing to the limited number of shedding events during the study. There were no obvious relationships with the majority of oxidative or adrenal stress markers, with the exception of 8-OHdG, suggesting cellular DNA damage might play a role in viral shedding processes associated with that disease in elephant calves. It is important to note that herpesvirus shedding itself does not necessarily equate to disease in the shedder, as it frequently occurs without any overt clinical signs. Rather, measures of variations in specific biomarkers associated with shedding might provide insight into the susceptibility of elephants to more severe primary infections. Interestingly, all studies to date measuring adrenal glucocorticoid (GC) activity in captive Thai elephants have found higher concentrations during the winter, which also is the high tourist season. The finding of the opposite pattern in this study, which was during the COVID-19 international tourism ban, suggests increases in GCs in earlier studies might have been due to tourist presence and associated activities, which could have welfare implications, or simply reflect more stimulation when people are present. Thus, this study serves as a nice control to examine health parameters in elephants unaffected by tourist activities, setting up additional studies to examine these same parameters once tourism resumes.

### 4.1. Oxidant markers

For ROS, adult elephants had overall higher concentrations than calves. This finding is consistent with a previous study on Asian elephants by Kosaruk et al. (14); however, the reason is unclear. It is possible that captive adult elephants are exposed to factors that contribute to higher ROS productions compared to calves, such as tourist activities and breeding. However, our study was conducted during the COVID-19 tourism ban, so that could not account for this pattern. Alternatively, dissimilar ROS activity could be attributable to differences in growth and development processes between calves and adults. Studies conducted in humans and birds showed that both young and aged individuals tend to have higher ROS concentrations compared to middle-aged groups (50, 51). ROS in young individuals is important for normal growth and development (50, 52), whereas high ROS concentrations in older individuals are associated with age-related diseases and conditions (53). Regarding seasonality, concentrations of ROS trended higher during the summer in both age groups, which is comparable to our earlier findings in captive elephants (14). In cattle, elevated ROS also were observed during periods of high ambient temperature and THI in South Asia (38, 54). In northern Thailand, summer is characterized by dry, warm conditions, with maximum temperatures reaching up to 40°C with relative humidity around 60%. Cattle are susceptible to heat stress when the THI exceeds 72 (55, 56), leading to increased ROS production and oxidative stress, and so this could be a stressor for captive elephants as well. However, elephants may be more tolerant of hot weather compared to cattle because of more darkly pigmented skin (14), which acts as a physical barrier, perhaps reducing the impact of oxidative stress (38). The small magnitude of change observed might suggest a mitigation of ROS activity throughout the year in this population of elephants.

For MDA, no age effect was observed, which aligns with previous research conducted on Asian elephants (14), as well as other species such as chimpanzees (57), horses (58), and humans (59). Regarding seasonal patterns, MDA concentrations remained relatively stable throughout the year except for 3 months during the rainy season (June, July, August) when lower concentrations were observed. A previous study in elephants showed no seasonal effect on MDA based on seasonal calculations (14), and although not significant, the overall mean was higher during the summer season. Studies in Asian cattle reported higher MDA concentrations during the summer, which also had the highest THI (40, 48), again demonstrating how cattle are susceptible to heat stress (56). In addition to summer, elephants in this study had higher MDA concentrations in the winter. The grasses that constitute the elephants’ diets tend to grow rapidly during the rainy season (60), so harvesting young grass may result in higher antioxidant content compared to dry season grasses that are usually harvested at a more mature stage (lower antioxidants) and could contribute to higher MDA in late rainy/winter season (61). Thus, the presence of higher antioxidants inhibiting the activity of free radicals (62, 63) could explain the lower MDA concentrations observed during this period.

For 8-OHdG, there was an age difference, with calves displaying higher concentrations than adults. Although there is a widely accepted connection between oxidative stress, DNA damage, and aging (29, 30), findings for 8-OHdG have been varied. With respect to aging, reports have shown no change (64) or a decrease (65) in 8-OHdG in humans, with increases observed in dogs (66), and decreases in ungulates (67), and chimpanzees (57). Decreases in 8-OHdG with aging may be attributed to reduced energy metabolism, as suggested in humans and ungulates studies (65, 67). For seasonality, elevated concentrations of 8-OHdG were found in elephants during the warmer months (March and May), aligning with studies in humans (68–70) showing higher 8-OHdG in summer to cope with high ambient temperature-induced oxidative stress. However, this contrasts with human studies in Netherlands (71) and China (72), which reported no seasonal effect on 8-OHdG, thus indicating possible environmental effects. A number of studies have demonstrated increased 8-OHdG responses to air pollution exposure (69, 73, 74). Summer months in northern Thailand are characterized by poor air quality due in part to crop burning, vehicular emissions, and temperature inversions that favor the stagnation of air (75, 76). Thus, heightened air pollution exposure could be leading to oxidative stress. If so, this study may be the first to demonstrate an impact of air pollution on biological functioning in captive elephants, which deserves further investigation.

### 4.2. Antioxidant markers

For serum albumin, adults had higher concentrations compared to calves, not unlike a previous study (14). By contrast, in humans, serum albumin concentrations peak at around 20 years and gradually decrease with age (77), potentially due to liver degeneration, as that is the primary site of albumin production (78), or to protein intake reduction, known as geriatric anorexia (79). Those conditions are unlikely causes of high albumin in elephants, but rather could be related to hydration. During the COVID-19 pandemic, both adults and calves were kept in confined spaces (80), which limited their activity and access to water. The highest albumin concentration (3.57 ± 0.27 g/dL) was in a 9 year-old calf (E13) that was mostly tethered (>16 h a day) and only occasionally offered water. Hydration status could also be related to seasonal patterns in water content of grasses, being higher during the rainy season in Thailand (61, 81). Dietary intake of protein also can increase albumin concentrations in humans (82, 83). The diet of elephants consists primarily of grasses (e.g., Napier grass), which have a relatively low protein content (<10%) (61, 84). Thus, the combination of low-protein foodstuffs with a higher fluid load during the rainy season may contribute to the relatively lower serum albumin concentrations observed during this period.

For GPx, no differences were observed between age groups, similar to studies on other species including mice (85), seabirds (86), goats (87), and Asian elephants (14). Although in humans, GPx concentrations decrease with age, especially after 65 years (88), the age range of elephants in this study was not broad enough to fully examine this effect. Regarding the seasonal pattern of GPx, activity was highest during the late summer to mid-rainy season (May to August), which contrasts with a previous study on elephants that showed no seasonal effect (14). Studies on deer and cattle have indicated that GPx activity is highest during the summer months, which can be attributed to the need to mitigate the impact of heat stress (39, 89, 90). In this study, the THI showed relatively constant values from March to November, indicating it alone may not explain the observed increase in GPx activity. Further investigations are necessary to identify other potential factors contributing to the seasonal variation in GPx activity among elephants.

For catalase, no age effect was found, consistent with previous studies of cattle (91), horses (92, 93), and also elephants (14). However, a human study by Casado and López-Fernández (94) found higher catalase activity in newborns (infants to 3 years) and the elderly (over 70 years). Supportive of that is the finding that the youngest elephant in this study (1 year and 9 months) had the highest catalase concentrations overall (18.80 ± 7.58 U/mL), although more animals across a broader age range are needed to confirm an age effect on this marker. Annual patterns showed the lowest concentrations between September and November, which marks a transition from rainy to winter seasons. Concentrations of 8-OHdG also were lower during those months, but it remains unclear what the significance of this seasonal effect is on elephant health.

### 4.3. Stress markers

For serum cortisol, no age effect was found in this study, which differs from previous studies that found concentrations can increase with age in some zoo-housed bull elephants (95, 96). No annual change in serum cortisol was observed, and the concentrations remained relatively stable throughout the year. A previous study of salivary cortisol reported an annual pattern for tourist camp elephants in Thailand (2), with higher concentrations exhibited during the winter.

For fGCM concentrations, there was no age effect similar to that previously reported in Thailand (14) and Myanmar (97). However, a notable finding was that the seasonal pattern of fGCM concentrations differed significantly from previous studies of this population that clearly showed higher rather than lower fGCM during the winter (2, 42, 98, 99). Because winter is also the high tourist season when elephants participate in activities like riding, bathing and feeding of high energy fruits (e.g., sugarcane, banana), it had not been possible to tease apart seasonal versus tourist effects on fGCM excretion. This study was conducted during the COVID-19 pandemic when all international flights were banned, and elephant tourist camps in Thailand closed (80). Thus, the results of this study strongly suggest it is tourist activities that are having a stimulatory effect on adrenal activity. The reason for higher fGCM between April and September could instead be due to temperature or THI effects, this is particularly relevant to elephants, as their low surface area-to-volume ratio and limited capacity for evaporating heat make them susceptible to overheating, potentially leading to heightened adrenal activity during these periods as suggested by Mumby et al. (100). It is unclear why serum cortisol and fGCM were not correlated, although that has been noted in other studies (101, 102). Serum reflects an immediate snapshot of circulating cortisol levels, and could be affected by the stress of blood sampling, whereas fGCM provides a summary of adrenocortical activity 24–48 h prior to sample collection (103, 104). Thus, minor fluctuations in circulating cortisol would not be evident in fecal profiles.

### 4.4. EEHV shedding

Previous studies have shown that EEHV-HD survivors can become shedders of the virus later in life, as it enters a latency stage (7, 43, 45, 105, 106). Our results confirmed elephants that survived EEHV infection intermittently shed the virus via saliva throughout the study period, Additionally, the same subtype of EEHV was detected in sheddings, consistent with the previous infection. Notably, this study also revealed that elephants without a history of EEHV or residing in camps with no previous EEHV cases still shed the virus, a finding noted before (11, 45). No seasonality was observed as EEHV shedding occurred throughout the year. This is consistent with a report by Yun et al. (10) that showed EEHV cases are found in every month of the year in Thailand.

The specific triggers for activating or shedding EEHV have yet to be clearly identified, but are presumed to be associated with stress events (7). Thus, stress-induced oxidative imbalances might play a role in EEHV shedding or reactivation of the virus (7, 14, 105). Considering high oxidant and low antioxidant activity makes animals more prone to disease (13, 33, 34), we expected to see significant patterns with EEHV shedding. However, our results were not consistent across elephants, possibly due to the infrequent occurrence of shedding events and limited sample numbers. Those with higher MDA concentrations were more likely to show EEHV shedding, but this was only observed in six elephants (E1, E2, E7, E8, E12, and E14). Unlike ROS and MDA, patterns of 8-OHdG were fairly consistent throughout the year, and only two elephants (E2 and E11) with high 8-OHdG exhibited EEHV shedding. Predicting EEHV shedding patterns based on albumin levels proved difficult, as both high and low concentrations were observed during viral shedding events. The shedding pattern for catalase, similar to albumin, was also challenging to clarify. Notably, in E12, high catalase concentrations from March to May corresponded to EEHV shedding, which also was associated with highly fluctuating ROS and MDA concentrations. These findings highlight the complexity of the relationship between oxidative stress markers and EEHV shedding. It appears that ROS and MDA may have a stronger association with viral shedding, while the relationship with 8-OHdG, albumin, GPx, and catalase requires further investigation.

### 4.5. Correlation matrix

There were significant associations among some of the oxidative, antioxidative and stress markers in this study. There were positive relationships between albumin and oxidative stress markers (ROS, MDA, and 8-OHdG), which was unexpected because higher concentrations of antioxidants are typically linked to lower oxidative stress levels (107, 108). However, serum albumin possesses a number of physiological properties that are not directly related to oxidative stress (109). Rather, the elevated serum albumin observed in this study could be a result of reduced fluid intake (110), which in turn may contribute to the modulation of other oxidant markers. The association between catalase and GPx activity was not surprising, as these two enzymatic antioxidants work to control hydrogen peroxide production by converting it to water (34, 111). The positive association between catalase and oxidant markers (ROS and 8-OHdG) was not unexpected, as increased catalase activity can be a compensatory mechanism for excessive production of ROS, as described in human and cattle studies where catalase positively correlated with ROS and 8-OHdG concentrations (112, 113).

For stress markers, as described above, no correlation was found between serum cortisol and fGCM concentrations, but high fGCM concentrations were associated with increased 8-OHdG concentrations, similar to a study conducted on zoo grizzly bears (114). That finding suggests a potential relationship between adrenal activity and rates of DNA damage. The environmental factor THI was positively associated with ROS and GPx in agreement with previous studies in other species (35, 38, 115). In addition, a higher THI was associated with increased fGCM concentrations, most likely due to higher ambient temperatures effects on adrenal activity (116, 117). This is the first study to reveal a seasonal environmental effect on fGCM in elephants, and provides compelling evidence that high concentrations during the winter season in prior studies is mostly due to elephant activities during the high tourist season (2, 42, 98).

Interestingly, the only relationship between oxidative stress markers and EEHV was a positive correlation between 8-OHdG and shedding (Ct). This finding suggests the presence of higher 8-OHdG concentrations may reflect activation of oxidative stress pathways as a result of EEHV infection, potentially playing a role in the pathogenesis of this disease. The precise mechanism by which increased 8-OHdG contributes to viral shedding remains to be fully elucidated, however, it is possible that DNA damage caused by the viral replication and shedding process triggers the activation of oxidative stress pathways, leading to the generation of ROS. While this current study did not find a direct association between ROS levels and viral shedding, it is important to note that oxidative stress encompasses a broader range of cellular responses beyond ROS production (13, 14, 22). Other mechanisms involving 8-OHdG could be involved, perhaps reflecting the presence of ongoing inflammation and cellular damage (29, 31) associated with viral shedding. The release of viral particles from infected cells can induce an immune response and inflammatory processes, which then can contribute to oxidative stress and subsequent DNA damage (118, 119). Further research is needed to unravel the precise mechanisms involved and explore the importance of monitoring 8-OHdG as a predictive marker for EEHV shedding and disease progression in captive Asian elephants. As mentioned earlier, shedding alone does not always correlate with disease signs. However, it might be possible that fluctuations in oxidative stress markers with or without shedding may indicate elephants’ vulnerability to severe primary infections, providing another analytical tool.

## 5. Conclusion

This study is the first to investigate seasonal patterns of biomarkers indicating oxidative stress in captive elephants in Thailand. The results revealed significant age effects on ROS and albumin, with adults showing higher concentrations than calves. Seasonal effects were clearly observed for several biomarkers; high values in summer for ROS, MDA, 8-OHdG, and albumin, and low values in the rainy/winter season for MDA, 8-OHdG, albumin, and catalase. Thus, there may be physiological adaptations in oxidative stress conditions due to seasonal or other environmental changes. Interestingly, the seasonal pattern of fGCM concentrations differed from a number of previous studies suggesting it is largely driven by tourist activities that mask a more subtle seasonal climate effect. Rather, without tourists during the COVID-19 lockdown, higher fGCM concentrations occurred during high THI months, indicating a stimulatory effect of high temperature and humidity on adrenal function in the absence of tourists. Continued research over an extended time period would offer further insights to confirm and refine the findings of this study. As previously reported, intermittent shedding of EEHV was observed through the year, and in this study, regardless of prior history or camp residency. The relationship between studied biomarkers and EEHV shedding was inconsistent, and not in a predictive way, likely due to the limited occurrence of shedding events in this study. The results highlight the complexity of this association, although its status as a genuine cause-and-effect relationship remains uncertain. Consequently, ongoing research and vigilant monitoring, utilizing larger sample sizes, are imperative to gain a more comprehensive understanding of this connection between oxidative stress and EEHV.

## Data availability statement

The original contributions presented in the study are included in the article/Supplementary material, further inquiries can be directed to the corresponding author.

## Ethics statement

The animal studies were approved by Animal Care and Use Committee, Faculty of Veterinary Medicine, Chiang Mai University, reference number S7/2564. The studies were conducted in accordance with the local legislation and institutional requirements. Written informed consent was obtained from the owners for the participation of their animals in this study.

## Author contributions

WK: Conceptualization, Formal analysis, Investigation, Validation, Writing – review & editing, Methodology, Visualization. JB: Conceptualization, Formal analysis, Investigation, Validation, Writing – original draft, Writing – review & editing, Data curation, Funding acquisition, Project administration, Resources, Supervision. PTn: Data curation, Formal analysis, Investigation, Writing – review & editing, Methodology. KP: Data curation, Formal analysis, Investigation, Writing – review & editing, Conceptualization, Resources, Supervision, Validation. VP: Conceptualization, Formal analysis, Investigation, Supervision, Validation, Writing – review & editing, Project administration, Software, Visualization. PTw: Writing – review & editing, Methodology. NK: Methodology, Writing – review & editing. WT: Methodology, Writing – review & editing. TJ: Methodology, Writing – review & editing. PM: Methodology, Writing – review & editing. CT: Writing – review & editing, Conceptualization, Data curation, Formal analysis, Funding acquisition, Investigation, Project administration, Resources, Supervision, Validation, Writing – original draft.

## Funding

The author(s) declare financial support was received for the research, authorship, and/or publication of this article. This study was supported by the Smithsonian Conservation Biology Institute (SCBI, United States) through a grant from the Shared Earth Foundation as part of a Memorandum of Understanding with Faculty of Veterinary Medicine, Chiang Mai University (R000032244) and the Elephant, Wildlife, and Companion Animals Research Group (1-2566), and WK was supported by a CMU Presidential scholarship.

## Conflict of interest

The authors declare that the research was conducted in the absence of any commercial or financial relationships that could be construed as a potential conflict of interest.

## Publisher’s note

All claims expressed in this article are solely those of the authors and do not necessarily represent those of their affiliated organizations, or those of the publisher, the editors and the reviewers. Any product that may be evaluated in this article, or claim that may be made by its manufacturer, is not guaranteed or endorsed by the publisher.
