# Supplementary material for: Seasonal patterns of oxidative stress markers in captive Asian elephants in Thailand and relationships to elephant endotheliotropic herpesvirus shedding
Source: Front Vet Sci. 2023 Sep 19;10:1263775. doi: 10.3389/fvets.2023.1263775 (PMC10546319; doi:10.3389/fvets.2023.1263775)
Supplement: Supplementary file 1 [file Table_1.DOCX]

Supplementary Material

# Supplementary Figures and Tables

## Supplementary Figures


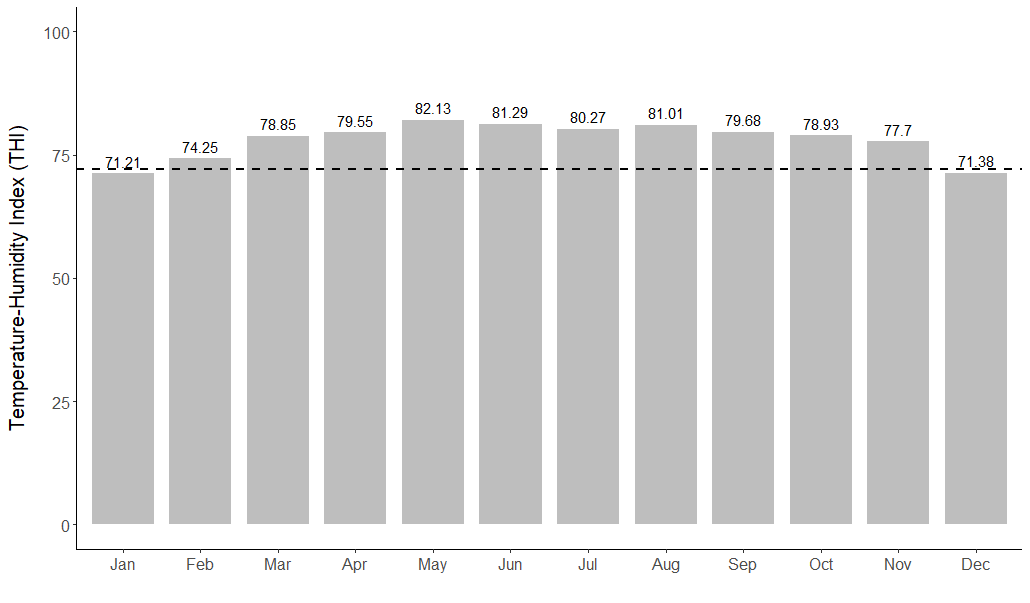

**Figure S1.** Monthly THI data during the study period. The dash line represented THI above 72, which is associated with heat stress in cattle (Mirzad et al., 2018).

**References**

Mirzad, A.N., Tada, T., Ano, H., Kobayashi, I., Yamauchi, T., Katamoto, H., 2018. Seasonal changes in serum oxidative stress biomarkers in dairy and beef cows in a daytime grazing system. J. Vet. 80, 20–27. doi:10.1292/jvms.17-0321


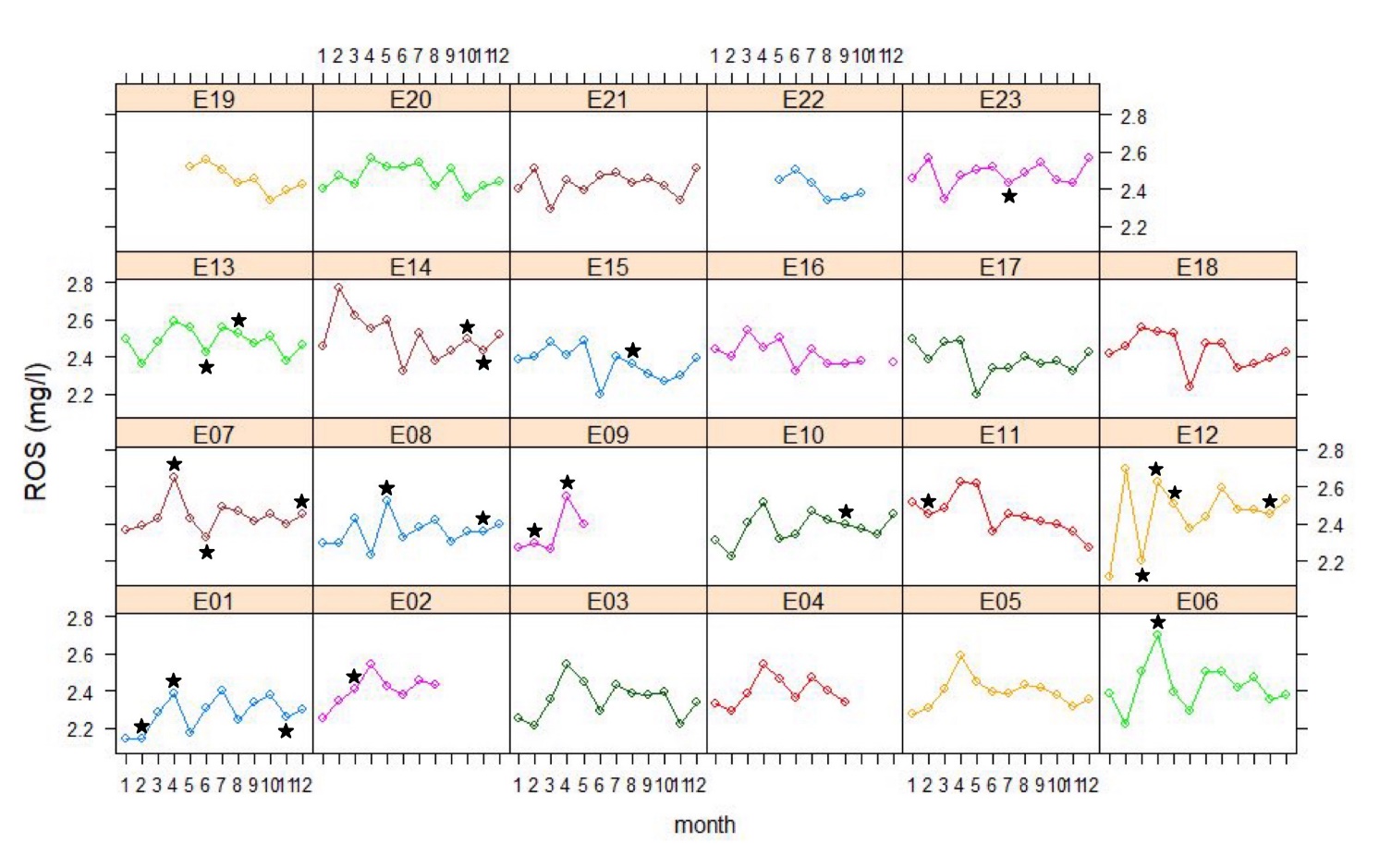


**Figure S2.** Line plot displays monthly pattern of reactive oxygen species (ROS) concentrations of individual elephants in this study. Different colors represent different elephants. Black star indicates detected shedding events. The numbers 1 to 12 on the x-axis represent each month respectively, from January to December.


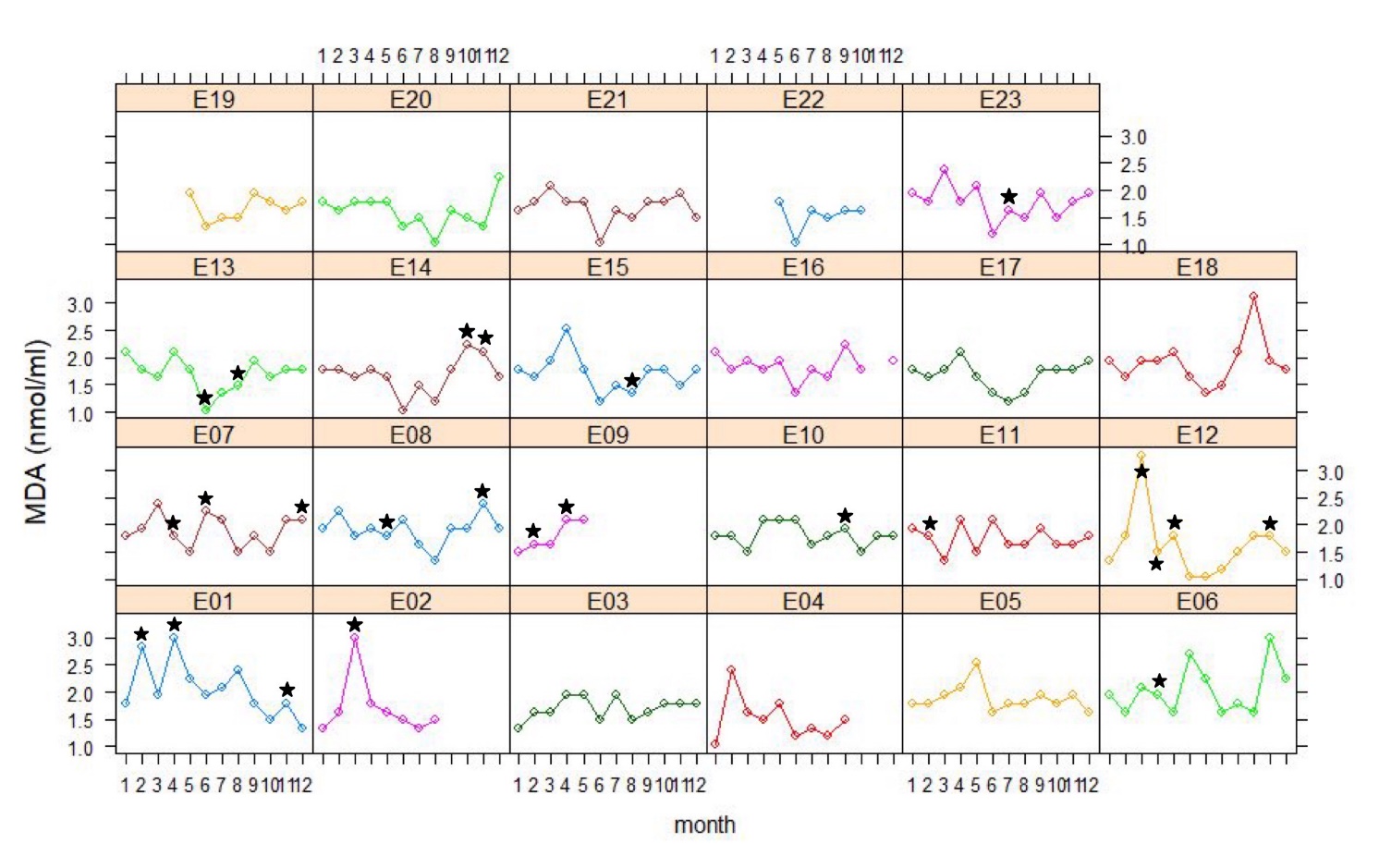


**Figure S3.** Line plot displays monthly pattern of malondialdehyde (MDA) concentrations of individual elephants in this study. Different colors represent different elephants. Black star indicates detected shedding events. The numbers 1 to 12 on the x-axis represent each month respectively, from January to December.


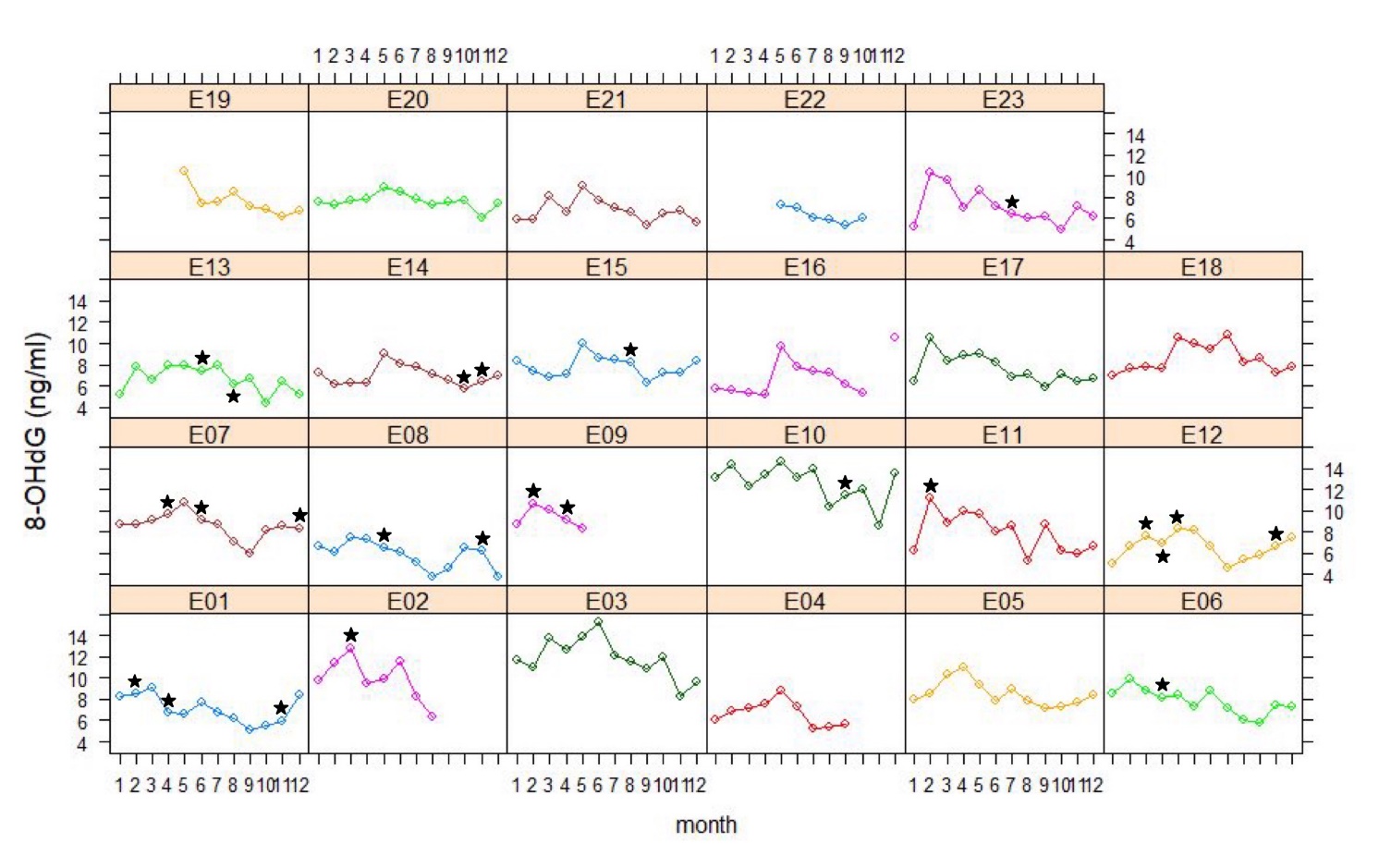


**Figure S4.** Line plot displays monthly pattern of 8-hydroxydeoxyguanosine (8-OHdG) concentrations of individual elephants in this study. Different colors represent different elephants. Black star indicates detected shedding events. The numbers 1 to 12 on the x-axis represent each month respectively, from January to December.


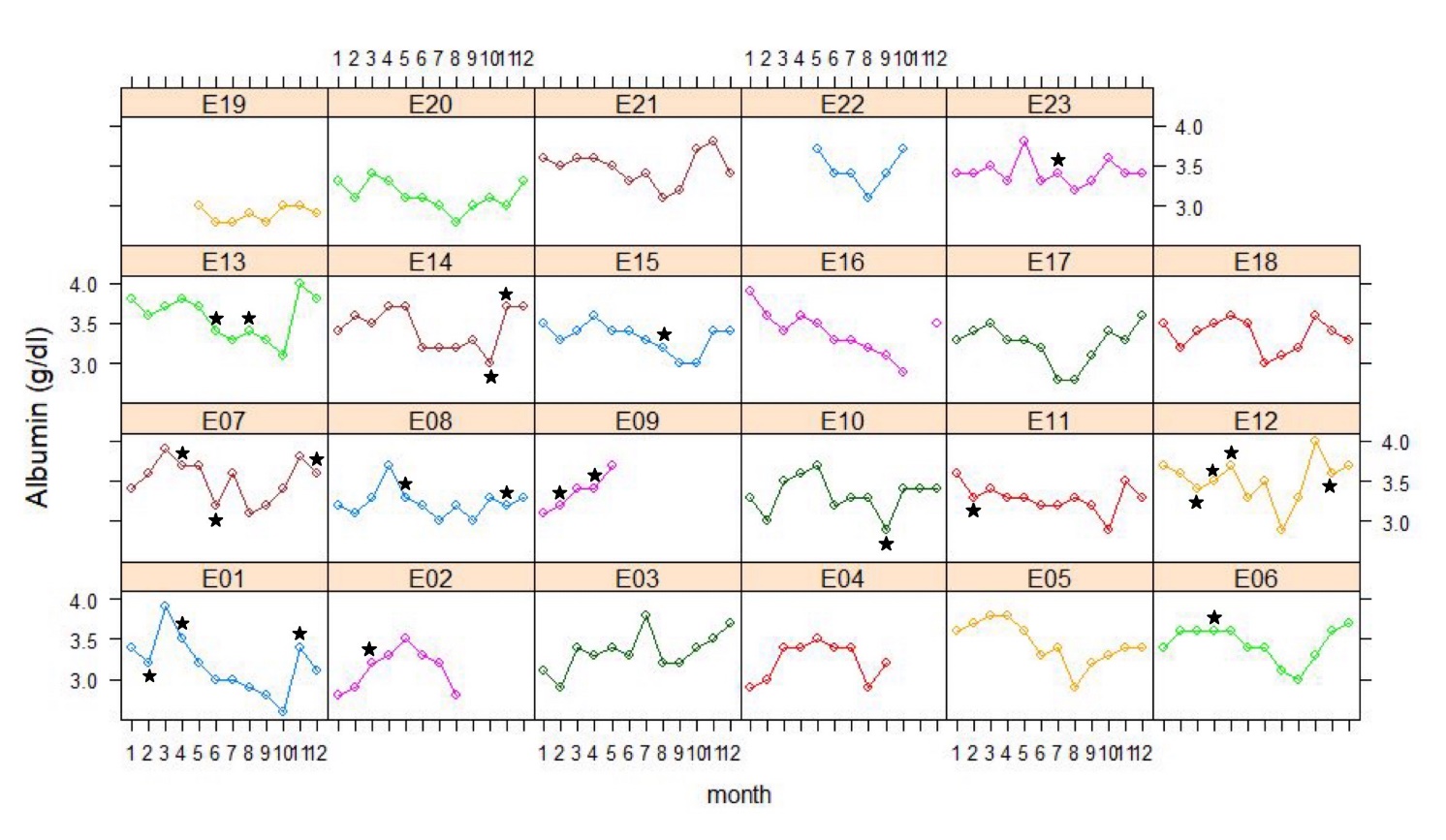


**Figure S5.** Line plot displays monthly pattern of serum albumin concentrations of individual elephants in this study. Different colors represent different elephants. Black star indicates detected shedding events. The numbers 1 to 12 on the x-axis represent each month respectively, from January to December.


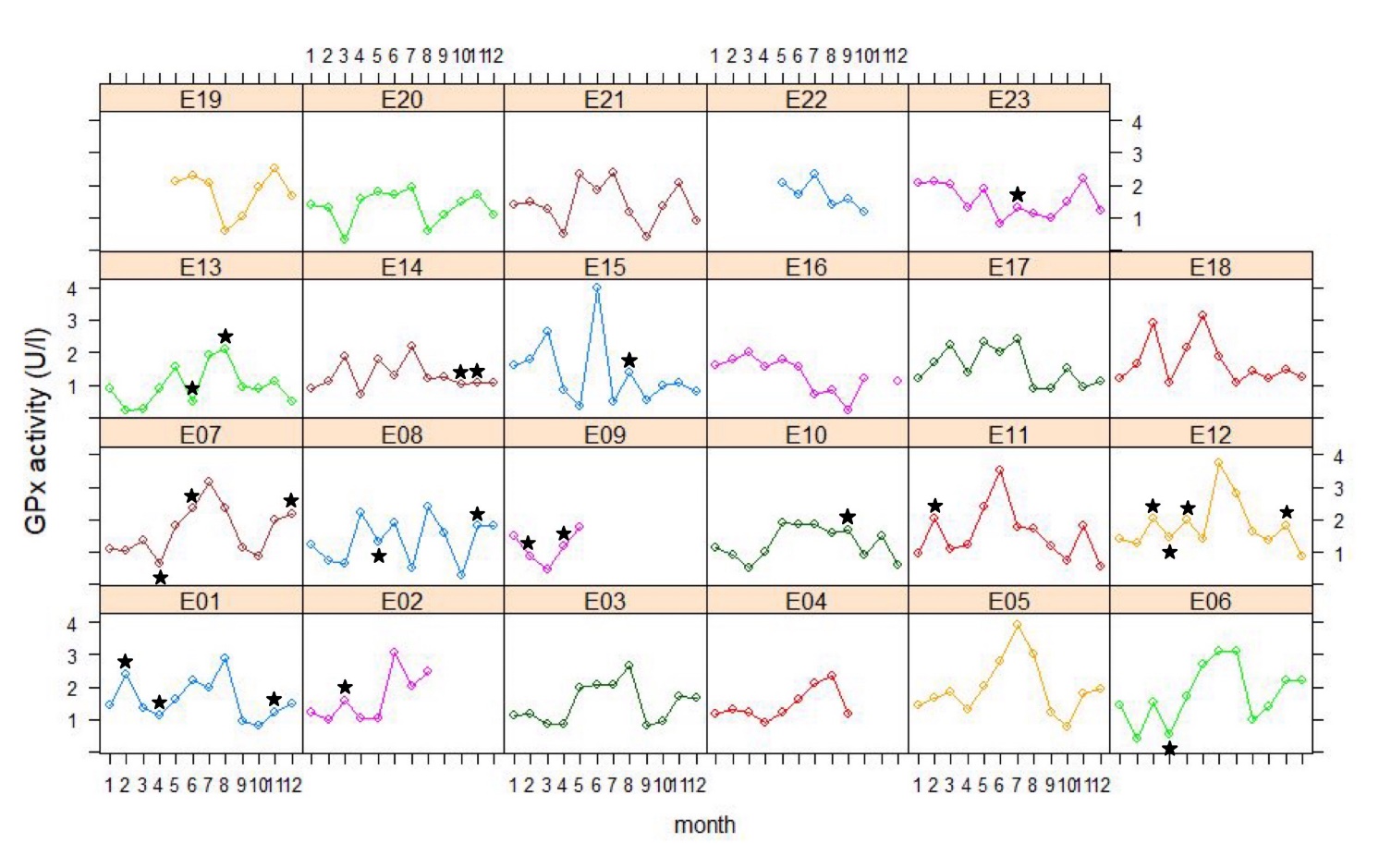


**Figure S6.** Line plot displays monthly pattern of glutathione peroxidase activity (GPx) of individual elephants in this study. Different colors represent different elephants. Black star indicates detected shedding events. The numbers 1 to 12 on the x-axis represent each month respectively, from January to December.


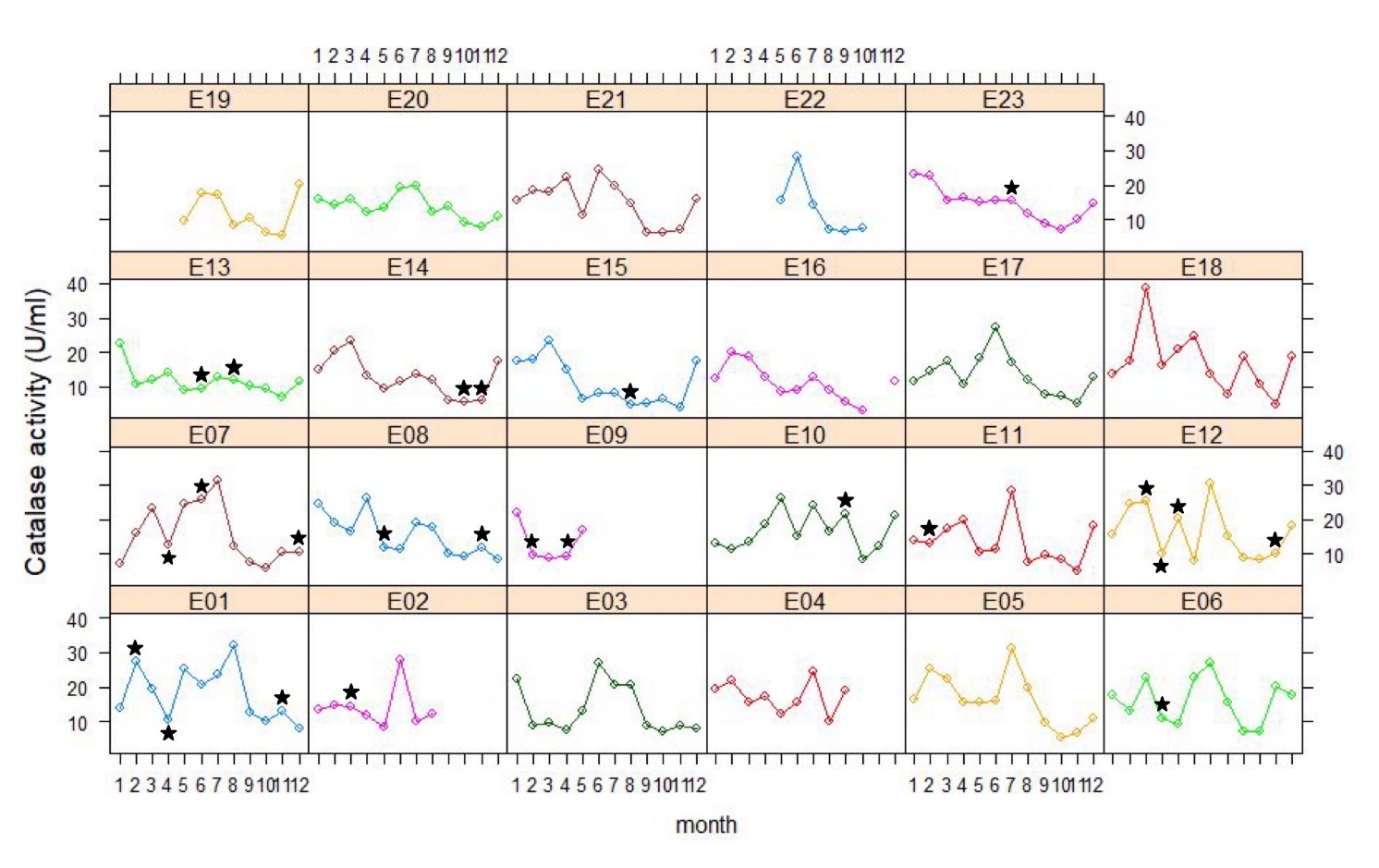


**Figure S7.** Line plot displays monthly pattern of catalase activities of individual elephants in this study. Different colors represent different elephants. Black star indicates detected shedding events. The numbers 1 to 12 on the x-axis represent each month respectively, from January to December.


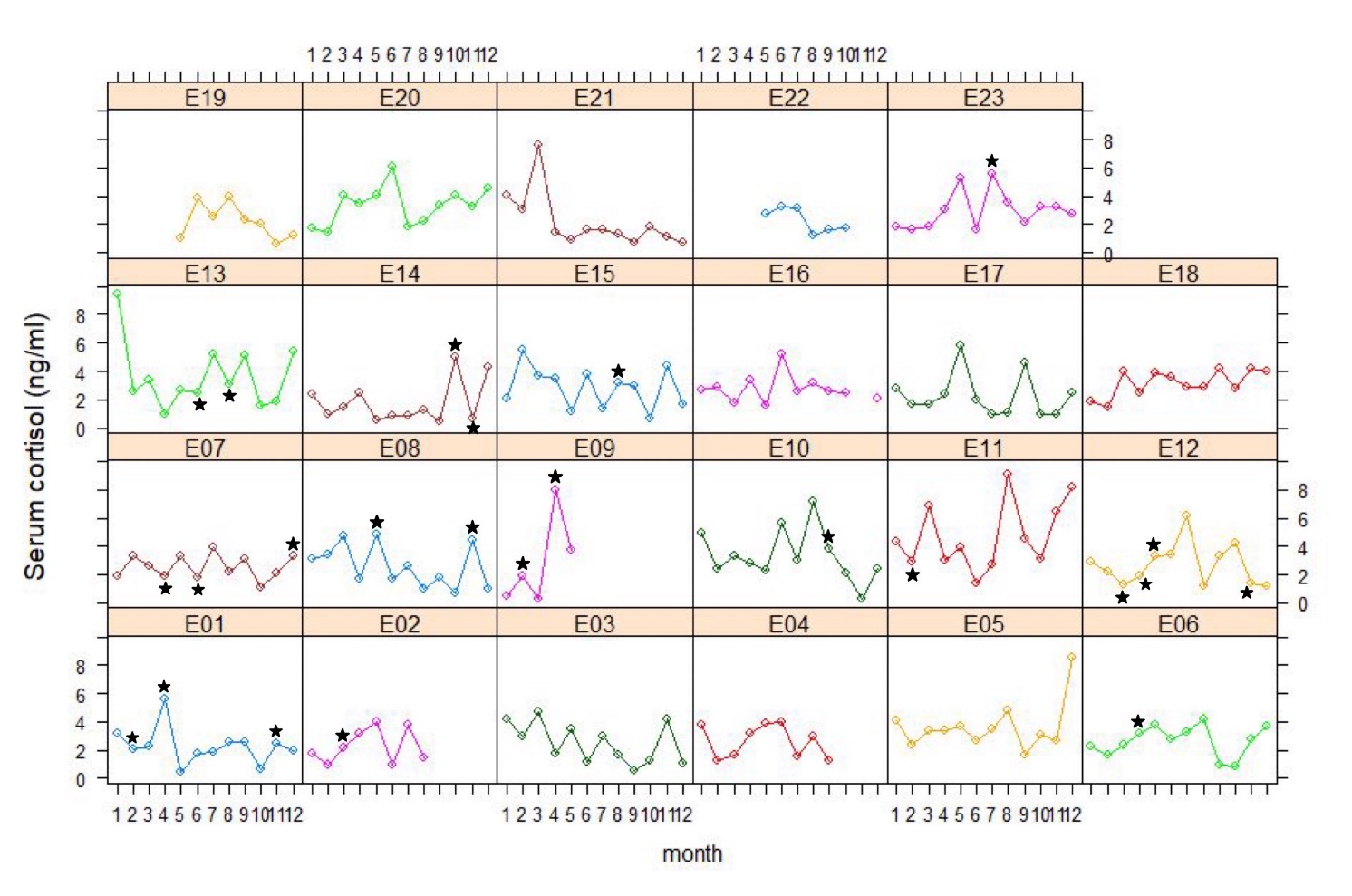


**Figure S8.** Line plot displays monthly pattern of serum cortisol concentrations of individual elephants in this study. Different colors represent different elephants. Black star indicates detected shedding events. The numbers 1 to 12 on the x-axis represent each month respectively, from January to December.


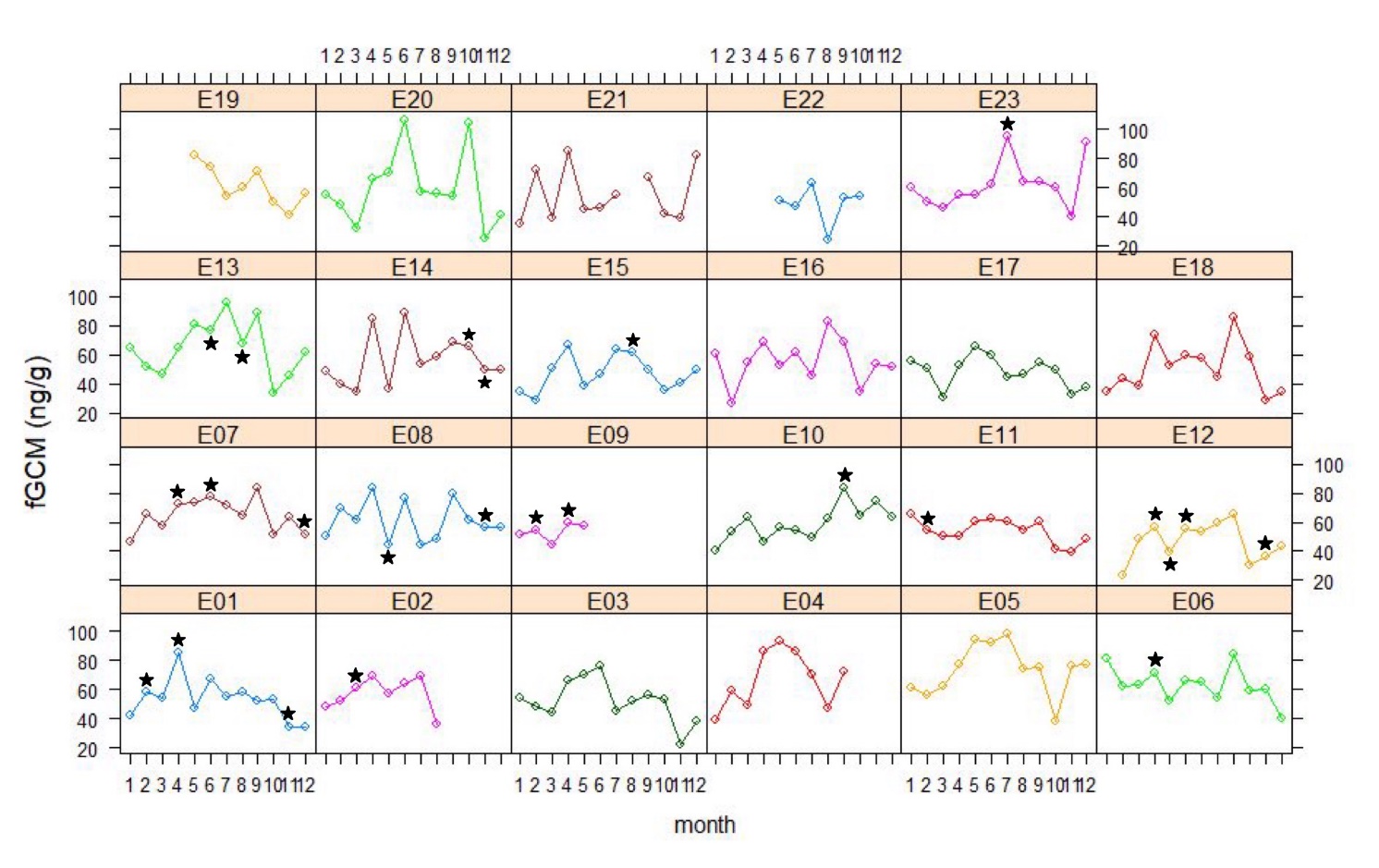


**Figure S9.** Line plot displays monthly pattern of fecal glucocorticoid metabolite (fGCM) concentrations of individual elephants in this study. Different colors represent different elephants. Black star indicates detected shedding events. The numbers 1 to 12 on the x-axis represent each month respectively, from January to December.

## Supplementary Tables

**Table S1.** General information about four EEHV-HD survived elephants in this study, which included current age, sex, and previous EEHV-HD history.

| **ID** | **Sex** | **Current age (in 2021)** | **Camp ID** | **Age at active EEHV-HD** | **Year** | **EEHV type detected** | **Initial clinical signs** |
| --- | --- | --- | --- | --- | --- | --- | --- |
| E9 | F | 5y 4m | F | 2y 4m | 2018 | 3/4 | Colic |
| E11 | M | 7y 10m | G | 3y | 2017 | 1B | Facial swollen |
| E12 | M | 2y 10m | D | 2y 7m | 2021 | 1A | Facial swollen, tongue hemorrhage |
| E13 | M | 9y 11m | G | 3y 5m | 2014 | 1A | Facial swollen |

EEHV-HD - elephant endotheliotropic herpesvirus-hemorrhagic disease.

**Table S2.** Generalized least square analysis of age groups, months, and interaction effects associated with oxidative stress markers.

| **Variables** | | **Reactive Oxygen Species** | | | **Malondialdehyde** | | | **8-Hydroxydeoxyguanosine** | | |
| --- | --- | --- | --- | --- | --- | --- | --- | --- | --- | --- |
|  |  | **Estimate** | **SEM** | **P-value** | **Estimate** | **SEM** | **P-value** | **Estimate** | **SEM** | **P-value** |
| **Age group** | Calves (≤8 years) | Reference |  |  | Reference |  |  | Reference |  |  |
|  | Adults (>8 years) | 0.126 | 0.039 | <0.01** | 0.241 | 0.126 | 0.057 | -1.980 | 0.797 | 0.014* |
| **Month** | January | Reference |  |  | Reference |  |  | Reference |  |  |
|  | February | 0.019 | 0.034 | 0.550 | 0.139 | 0.110 | 0.208 | 1.096 | 0.432 | 0.012* |
|  | March | 0.087 | 0.030 | <0.01** | 0.245 | 0.107 | 0.023* | 1.381 | 0.432 | <0.01** |
|  | April | 0.229 | 0.031 | <0.01** | 0.257 | 0.107 | 0.017* | 0.915 | 0.432 | 0.035* |
|  | May | 0.122 | 0.031 | <0.01** | 0.222 | 0.107 | 0.039* | 1.194 | 0.432 | <0.01** |
|  | June | 0.020 | 0.033 | 0.518 | 0.179 | 0.107 | 0.096 | 0.820 | 0.443 | 0.066 |
|  | July | 0.124 | 0.033 | <0.01** | 0.070 | 0.107 | 0.513 | 0.075 | 0.443 | 0.865 |
|  | August | 0.103 | 0.034 | <0.01** | -0.031 | 0.110 | 0.776 | -1.539 | 0.443 | <0.01** |
|  | September | 0.069 | 0.031 | 0.031* | 0.133 | 0.110 | 0.227 | -1.198 | 0.456 | <0.01** |
|  | October | 0.081 | 0.034 | 0.017* | 0.036 | 0.118 | 0.760 | -0.824 | 0.471 | 0.081 |
|  | November | 0.022 | 0.034 | 0.512 | 0.373 | 0.118 | <0.01** | -1.254 | 0.471 | <0.01** |
|  | December | 0.126 | 0.032 | 0.045* | 0.122 | 0.113 | 0.283 | -0.349 | 0.471 | 0.459 |
| **Age x Month** | Calves x January | Reference |  |  | Reference |  |  | Reference |  |  |
|  | Adult x February | 0.018 | 0.047 | 0.690 | -0.296 | 0.164 | 0.073 | -0.002 | 0.660 | 0.998 |
|  | Adult x March | -0.060 | 0.046 | 0.196 | -0.219 | 0.162 | 0.178 | -0.483 | 0.660 | 0.465 |
|  | Adult x April | -0.171 | 0.046 | <0.01** | -0.180 | 0.162 | 0.268 | -0.266 | 0.660 | 0.687 |
|  | Adult x May | -0.087 | 0.045 | 0.055 | -0.256 | 0.159 | 0.108 | 1.521 | 0.645 | 0.019* |
|  | Adult x June | -0.063 | 0.045 | 0.168 | -0.827 | 0.159 | <0.01** | 0.731 | 0.653 | 0.264 |
|  | Adult x July | -0.101 | 0.045 | 0.028* | -0.445 | 0.159 | <0.01** | 1.031 | 0.653 | 0.116 |
|  | Adult x August | -0.131 | 0.046 | <0.01** | -0.450 | 0.162 | <0.01** | 2.453 | 0.653 | <0.01** |
|  | Adult x September | -0.095 | 0.046 | 0.040* | -0.140 | 0.161 | 0.384 | 1.266 | 0.661 | 0.056 |
|  | Adult x October | -0.129 | 0.048 | <0.01** | -0.163 | 0.168 | 0.332 | 0.835 | 0.672 | 0.215 |
|  | Adult x November | -0.091 | 0.049 | 0.062 | -0.488 | 0.170 | <0.01** | 1.320 | 0.688 | 0.056 |
|  | Adult x December | -0.057 | 0.047 | 0.228 | -0.169 | 0.165 | 0.306 | 0.968 | 0.678 | 0.155 |

Asterisks indicate significant differences at P<0.05 (*) and P<0.01 (**).

**Table S3.** Generalized least square analysis of age group, month, and interaction effects associated with antioxidant stress markers.

| **Variables** | | **Serum albumin** | | | **Glutathione peroxidase** | | | **Catalase** | | |
| --- | --- | --- | --- | --- | --- | --- | --- | --- | --- | --- |
|  |  | **Estimate** | **SEM** | **P-value** | **Estimate** | **SEM** | **P-value** | **Estimate** | **SEM** | **P-value** |
| **Age group** | Calves (≤8 years) | Reference |  |  | Reference |  |  | Reference |  |  |
|  | Adults (>8 years) | 0.256 | 0.102 | 0.013* | 0.186 | 0.257 | 0.471 | -0.105 | 2.339 | 0.964 |
| **Month** | January | Reference |  |  |  |  |  |  |  |  |
|  | February | 0.0002 | 0.076 | 0.997 | -0.153 | 0.235 | 0.515 | -1.146 | 2.170 | 0.598 |
|  | March | 0.273 | 0.074 | <0.01** | -0.114 | 0.228 | 0.618 | -0.079 | 2.114 | 0.970 |
|  | April | 0.225 | 0.074 | <0.01** | -0.135 | 0.229 | 0.555 | -2.087 | 2.115 | 0.325 |
|  | May | 0.225 | 0.074 | <0.01** | 0.449 | 0.229 | 0.051 | -1.394 | 2.117 | 0.511 |
|  | June | -0.022 | 0.074 | 0.768 | 1.060 | 0.229 | <0.01** | 1.532 | 2.117 | 0.470 |
|  | July | 0.069 | 0.074 | 0.354 | 1.130 | 0.229 | <0.01** | 7.840 | 2.117 | <0.01** |
|  | August | -0.187 | 0.076 | 0.014* | 1.201 | 0.234 | <0.01** | -0.318 | 2.168 | 0.884 |
|  | September | -0.195 | 0.076 | 0.011* | -0.027 | 0.235 | 0.907 | -5.334 | 2.170 | 0.014* |
|  | October | 0.052 | 0.082 | 0.527 | -0.366 | 0.250 | 0.145 | -9.240 | 2.312 | <0.01** |
|  | November | 0.206 | 0.082 | 0.012* | 0.516 | 0.250 | 0.040* | -5.321 | 2.312 | 0.022* |
|  | December | 0.163 | 0.079 | 0.039* | 0.211 | 0.242 | 0.383 | -3.360 | 2.234 | 0.134 |
| **Age x Month** | Calves x January | Reference |  |  | Reference |  |  | Reference |  |  |
|  | Adult x February | -0.135 | 0.114 | 0.235 | 0.196 | 0.351 | 0.576 | 1.988 | 3.244 | 0.540 |
|  | Adult x March | -0.330 | 0.112 | <0.01** | 0.420 | 0.346 | 0.227 | 3.923 | 3.207 | 0.222 |
|  | Adult x April | -0.249 | 0.112 | 0.027* | -0.198 | 0.347 | 0.569 | 0.309 | 3.208 | 0.923 |
|  | Adult x May | -0.272 | 0.110 | 0.014* | 0.057 | 0.339 | 0.867 | -2.725 | 3.132 | 0.385 |
|  | Adult x June | -0.243 | 0.110 | 0.028* | -0.611 | 0.339 | 0.073 | -0.373 | 3.132 | 0.905 |
|  | Adult x July | -0.425 | 0.110 | <0.01** | -0.789 | 0.339 | 0.021* | -9.479 | 3.132 | <0.01** |
|  | Adult x August | -0.239 | 0.112 | 0.035* | -1.524 | 0.346 | <0.01** | -6.550 | 3.203 | 0.042* |
|  | Adult x September | -0.178 | 0.111 | 0.111 | -0.473 | 0.343 | 0.169 | -2.256 | 3.169 | 0.477 |
|  | Adult x October | -0.332 | 0.116 | <0.01** | 0.239 | 0.358 | 0.505 | -0.452 | 3.302 | 0.891 |
|  | Adult x November | -0.266 | 0.118 | 0.025* | -0.384 | 0.362 | 0.290 | -4.973 | 3.343 | 0.138 |
|  | Adult x December | -0.244 | 0.114 | 0.034* | -0.581 | 0.351 | 0.099 | 1.947 | 3.246 | 0.549 |

Asterisks indicate significant differences at P<0.05 (*) and P<0.01 (**).

**Table S4.** Generalized least square analysis of age group, month, and interaction effects associated with two stress biomarkers.

| **Variables** | | **Serum cortisol** | | | **Fecal Glucocorticoid Metabolites** | | |
| --- | --- | --- | --- | --- | --- | --- | --- |
|  |  | **Estimate** | **SEM** | **P-value** | **Estimate** | **SEM** | **P-value** |
| **Age group** | Calves (≤8 years) | Reference |  |  | Reference |  |  |
|  | Adults (>8 years) | 0.093 | 0.722 | 0.897 | -1.846 | 6.596 | 0.779 |
| **Month** | January | Reference |  |  | Reference |  |  |
|  | February | -0.810 | 0.640 | 0.207 | 1.950 | 5.707 | 0.732 |
|  | March | -0.122 | 0.640 | 0.848 | 2.629 | 5.538 | 0.635 |
|  | April | 0.215 | 0.640 | 0.737 | 14.741 | 5.558 | <0.01** |
|  | May | 0.278 | 0.640 | 0.665 | 11.024 | 5.556 | 0.048* |
|  | June | -0.623 | 0.655 | 0.343 | 18.825 | 5.562 | <0.01** |
|  | July | 0.118 | 0.655 | 0.857 | 10.002 | 5.562 | 0.073 |
|  | August | 0.382 | 0.655 | 0.560 | 2.260 | 5.689 | 0.691 |
|  | September | -0.774 | 0.673 | 0.251 | 18.825 | 5.711 | <0.01** |
|  | October | -1.239 | 0.694 | 0.076 | -2.508 | 6.101 | 0.681 |
|  | November | -0.182 | 0.694 | 0.793 | -2.081 | 6.100 | 0.733 |
|  | December | 0.342 | 0.694 | 0.622 | -1.846 | 6.596 | 0.779 |
| **Age x Month** | Calves x January | Reference |  |  | Reference |  |  |
|  | Adult x February | -0.056 | 0.978 | 0.955 | -6.625 | 8.518 | 0.438 |
|  | Adult x March | 0.178 | 0.978 | 0.856 | -11.535 | 8.406 | 0.171 |
|  | Adult x April | -0.876 | 0.978 | 0.371 | 3.662 | 8.418 | 0.664 |
|  | Adult x May | -0.758 | 0.954 | 0.427 | -3.471 | 8.231 | 0.674 |
|  | Adult x June | 0.553 | 0.964 | 0.567 | -2.532 | 8.235 | 0.759 |
|  | Adult x July | -0.710 | 0.964 | 0.462 | 2.441 | 8.235 | 0.767 |
|  | Adult x August | -1.122 | 0.964 | 0.245 | 4.515 | 8.425 | 0.592 |
|  | Adult x September | 0.330 | 0.976 | 0.735 | -2.814 | 8.337 | 0.736 |
|  | Adult x October | 0.458 | 0.991 | 0.644 | 5.395 | 8.708 | 0.536 |
|  | Adult x November | -0.772 | 1.016 | 0.448 | -8.798 | 8.687 | 0.312 |
|  | Adult x December | -0.614 | 1.001 | 0.540 | 6.608 | 8.541 | 0.439 |

Asterisks indicate significant differences at P<0.05 (*) and P<0.01 (**).
